# Exploring the potential and limitations of deep learning and explainable AI for longitudinal life course analysis

**DOI:** 10.1186/s12889-025-22705-4

**Published:** 2025-04-24

**Authors:** Helen Coupland, Neil Scheidwasser, Alexandros Katsiferis, Megan Davies, Seth Flaxman, Naja Hulvej Rod, Swapnil Mishra, Samir Bhatt, H. Juliette T. Unwin

**Affiliations:** 1 Section of Epidemiology, Department of Public Health, University of Copenhagen, Copenhagen, Denmark; 2 Copenhagen Health Complexity Center, Department of Public Health, University of Copenhagen, Copenhagen, Denmark; 3 Department of Computer Science, University of Oxford, Oxford, UK; 4 Saw Swee Hock School of Public Health & Institute of Data Science, National University of Singapore, Singapore, Singapore; 5 MRC Centre for Global Infectious Disease Analysis, Imperial College, London, UK; 6 School of Mathematics, University of Bristol, Bristol, UK

**Keywords:** Deep learning, Life course epidemiology, Explainable artificial intelligence

## Abstract

**Background:**

Understanding the complex interplay between life course exposures, such as adverse childhood experiences and environmental factors, and disease risk is essential for developing effective public health interventions. Traditional epidemiological methods, such as regression models and risk scoring, are limited in their ability to capture the non-linear and temporally dynamic nature of these relationships. Deep learning (DL) and explainable artificial intelligence (XAI) are increasingly applied within healthcare settings to identify influential risk factors and enable personalised interventions. However, significant gaps remain in understanding their utility and limitations, especially for sparse longitudinal life course data and how the influential patterns identified using explainability are linked to underlying causal mechanisms.

**Methods:**

We conducted a controlled simulation study to assess the performance of various state-of-the-art DL architectures including CNNs and (attention-based) RNNs against XGBoost and logistic regression. Input data was simulated to reflect a generic and generalisable scenario with different rules used to generate multiple realistic outcomes based upon epidemiological concepts. Multiple metrics were used to assess model performance in the presence of class imbalance and SHAP values were calculated.

**Results:**

We find that DL methods can accurately detect dynamic relationships that baseline linear models and tree-based methods cannot. However, there is no one model that consistently outperforms the others across all scenarios. We further identify the superior performance of DL models in handling sparse feature availability over time compared to traditional machine learning approaches. Additionally, we examine the interpretability provided by SHAP values, demonstrating that these explanations often misalign with causal relationships, despite excellent predictive and calibrative performance.

**Conclusions:**

These insights provide a foundation for future research applying DL and XAI to life course data, highlighting the challenges associated with sparse healthcare data, and the critical need for advancing interpretability frameworks in personalised public health.

**Supplementary Information:**

The online version contains supplementary material available at 10.1186/s12889-025-22705-4.

## Introduction

The complexity of human health trajectories is shaped by the intricate interplay of biological, environmental, and social factors across the individuals’ lifespans [1]. Life course epidemiology seeks to understand these dynamics, revealing how early-life exposures, cumulative stressors, and critical developmental periods influence disease risk and health outcomes [2–6]. For instance, early-life adversities, such as childhood trauma or poverty, have been linked to adult-onset diseases, with the risk depending heavily on the timing, intensity and interplay with other exposures [7, 8]. With the increasing availability of large-scale longitudinal datasets, particularly those derived from national healthcare registers, researchers now have an unprecedented opportunity to examine health trajectories over the life course. These datasets are comprised of individual-level exposures recorded over a long time period, ranging from adverse childhood experiences (ACEs) to hospital records and education attainment, enabling the investigation of how risks accumulate and interact over time [1, 3]. However, traditional analytical methods, such as regression models and risk scoring, often fail to capture the full extent of the non-linear, multi-factorial relationships inherent in these processes [9, 10]. These methods rely on assumptions of linearity, independence, and population-level homogeneity, often leading to oversimplified interpretations that mask individual-level variations and temporal nuances [11]. As a result, the full richness of life course data remains underutilised, limiting the ability to inform targeted public health interventions.

Recent advancements in deep learning (DL) have allowed these models to overcome the limitations of traditional models in many fields. Within various healthcare applications, these models have proven to accurately predict disease risk for multiple outcomes using electronic health records (EHRs), including cardiovascular disease, cancer and diabetes [12–14]. They are particularly suited to these applications because of their ability to autonomously learn complex patterns and relationships directly from data, without requiring the pre-specification of their form or depending on strong assumptions [15]. Additionally, DL architectures, such as recurrent neural networks (RNNs) and convolutional neural networks (CNNs), are uniquely equipped to handle multivariate time-series (MTS) data, preserving temporal information that is often lost in traditional methods [16]. These qualities mean that DL models have the potential to transform life course epidemiology and the insights that can be gained from these vast datasets [17]. With the appropriate application of these methods, it may be possible to improve the accuracy of individual risk predictions, identify new risk factors, and find sensitive periods during which interventions are more effective. However, there is still a substantial amount of development required in order for them to be reliably and ethically implemented within life course epidemiology.

Whilst DL models have performed well in many other applications, there have been no studies examining the effectiveness of these models in life course epidemiology tasks. Moreover, although DL architectures designed for MTS data, such as RNNs and Transformers, have outperformed traditional methods in many contexts, some studies have found evidence of epidemiological settings where DL models perform worse [18, 19]. The application of DL to life course data remains limited, with significant gaps in understanding how these models perform under the unique constraints of longitudinal datasets, including sparsity and confounding. Many DL models have become state-of-the-art in specific applications, however, there is limited knowledge on which architectures are best suited to life course analysis. For instance, InceptionTime has performed well in physiological signal classification, such as ECG and EEG analysis [20, 21], and Transformers have revolutionised natural language processing (NLP) by achieving superior performance in tasks such as machine translation and text generation [22, 23]. While some models excel in capturing certain data characteristics, such as sparsity or temporal complexity, their relative performance can vary widely across different datasets and tasks. Understanding which DL models are most appropriate for life course analysis remains a critical gap in the field.

Another critical challenge is the interpretability of DL models, which often function as “black boxes”, with limited insights into the specific temporal or inter-variable relationships that they identify [24]. Recent advances in explainable artificial intelligence (XAI) aim to address this issue, with methods like Shapley additive explanations (SHAP) emerging as popular tool [25–29]. Methods such as SHAP provide post hoc explanations by quantifying the contribution of each input feature to a model’s predictions [25]. In life course epidemiology, this capability is particularly valuable for identifying critical risk factors and key exposure periods that influence health outcomes, providing actionable insights for public health [27]. SHAP is now one of the most widely used XAI methods in healthcare with a recent surge in studies using it to identify potential risk factors and disease mechanisms [30–32]. However, XAI methods remain limited in their ability to establish causal relationships, an issue that is especially pronounced in time-series data where confounding and sparsity complicate interpretability [33]. Within epidemiology, establishing causal relationships is considered essential and is one of the significant knowledge gaps restricting the use of DL within this field [34]. Without established causal approaches for DL models, many researchers use XAI methods like SHAP to identify the potential causal mechanisms, even though the SHAP values only relate to the predictive influence of exposures. Misinterpreting predictive markers as causal mechanisms poses significant risks, underscoring the need for rigorous evaluation of XAI and how it is related to the underlying causal structures present in life course applications.

There are several key ways in which longitudinal life course data sets differ from MTS data. One particular challenge is the sparsity which is a defining characteristic of many life course datasets. Sparse data-characterised by irregular sampling, rare events, and high proportions of missing or zero entries-pose significant challenges for traditional methods. For example, ACEs or exposures to rare diseases often occur infrequently, creating datasets with high sparsity. While many DL models were initially developed for tasks like NLP or computer vision, many models have been adapted for application to MTS data which is distinct from life course data [35]. Although DL models are frequently claimed to excel in handling such sparsity, there is little systematic evidence supporting this advantage. In fact, sparsity is considered to be one of the key limitations preventing the wider use of DL methods in healthcare, alongside model opacity and data heterogeneity [36]. Instead, most studies on DL performance in sparse datasets are case-specific and lack generalisability, leaving a critical gap in the literature.

This study addresses these gaps by systematically evaluating the utility of DL models and XAI methods for life course analysis for the first time. Through a series of simulation experiments, we compare the ability of four state-of-the-art DL models, including MLSTM-FCN, LSTMAttention, ResNet and InceptionTime, to handle varying levels of sparsity, exposure complexity, and confounding. These models are benchmarked against traditional methods, including logistic regression and XGBoost, to provide a comprehensive understanding of their strengths and limitations [37]. Additionally, we use SHAP to examine the consistency and validity of model explanations, comparing SHAP-derived insights to the ground truth causal mechanisms embedded in the simulations.

Our findings represent three key contributions to the advancement of life course epidemiology. First, we evaluate whether there is a universal best-performing DL model for analysing different possible relationships in life course data or if model performance is inherently scenario-dependent. Second, this study evaluates the reliability of SHAP as an interpretability tool, highlighting its limitations in aligning with causal relationships and cautioning against its misuse for identifying disease mechanisms or risk factors. Third, it investigates the capability of DL models to handle sparse healthcare data, providing evidence for their effectiveness in contexts where traditional methods often fail. The findings not only offer practical guidance for researchers but also contribute to the broader discussion on integrating causality, interpretability, and predictive accuracy in AI-driven public health solutions. This work aims to advance the responsible and effective use of AI in healthcare, paving the way for a new DL framework for life course analysis that has the power to improve our understanding of disease risk trajectories.

## Methods

This section outlines the methodological approach to simulating data as well as the parameter tuning and model fitting procedures. Further technical details are provided in the supplementary materials for reproducibility.

### Data simulation

We used the simcausal [38] R package to generate a longitudinal dataset with a Directed Acyclic Graph (DAG) designed to reflect multiple exposure occurring over time as well as realistic temporal and inter-variable dependencies. The use of the simcausal package ensures interpretability by maintaining an explicit causal structure. While alternative methods, such as simulated data generation using Generative Adversarial Networks, are capable of replicating real-world datasets with high fidelity, they often lack the transparency and generalisability required for the analysis of causal mechanisms, which is central to this study. Parameters for the DAG were derived from a subset of the DANLIFE cohort as established in Davies et al. to ensure that the simulated data captures the important relationships present in life course data [39]. The simulated dataset includes 100,000 individuals, followed from birth until their 16 th birthday, incorporating both time-dependent (dynamic) and time-independent (stationary) exposures. Specifically, three time-independent covariates (maternal age at birth, parental diabetes status, and parental ethnicity) and three time-dependent exposures (counts of ACEs as defined by Rod et al. [8]) were included.

The simulated cohort differs from the original DANLIFE cohort in the way ACEs are represented, the original cohort includes a single ACE variable that represents fitted trajectory groups which is replaced by the three time series variables (Loss, SES and Dynamic) that were used by Davies et al. to create the trajectory groups in the simulated cohort, thereby allowing a study of temporal patterns. The stationary variables were simulated using categorical distributions with probabilities to match the DANLIFE cohort, given in Table S1. The time-dependent variables were modelled as zero-inflated Poisson distributions that depended on the values of the time-independent variables and their value at the previous time point. This methodology generated individual-level trajectories over time, enabling the exploration of complex interactions among variables throughout the lifespan. To evaluate how well the synthetic data reflects the real-world DANLIFE cohort, we have included detailed statistical comparisons in the supplementary materials in Figure S5.

Multiple binary outputs $y$ were generated from the input data $X$ using custom functions, designed to replicate four Life Course Patterns (LCPs) common within the literature: period, repeats, order, and timing (Table 1). The World Health Organization originally conceptualised LCPs to categorise how different exposures at various life stages may affect health [40]. The period conceptual model is based on research showing that there are certain time periods during which exposure events have greater impact on future health [3]. For instance, childhood exposure to passive smoking is more detrimental to lung function than the same exposure occurring in adulthood [41]. Critical periods represent periods during which exposures must occur to give rise to certain health consequences, such as the irreversible effects of alcohol exposure during the first trimester of pregnancy on the child’s organ development, leading to lifelong impacts on learning, behaviour, and predisposition to various chronic diseases [42]. In contrast, sensitive periods increase the likelihood of poor outcomes but do not guarantee them and other factor outside this period may also impact outcome likelihood. Furthermore, the accumulation-of-risk model illustrates how repeated exposures can compound risk over time, such as chronic poverty exacerbating respiratory issues like asthma [3, 43]. Another key life course paradigm is the timing of exposures. The relative timing of events can magnify their health impacts, for example, experiencing multiple adversities, such as unemployment and illness within a short period, may compound negative outcomes [44]. Finally, the temporal ordering of exposures has a substantial impact on the health trajectory [45]. For instance, an individual’s likelihood of a heart attack is greater if they have experienced depression previously [46].

**Table 1:** Definitions of the rules generating disease outcomes

LCP		Positive (%)	Definition
**Period**	1	2.96	At least one Loss exposure event in the first two years of life.
	2	2.27	At least two Loss exposure events in the first five years of life.
	3	1.68	Weighted sum of Loss exposures exceeds 1. Events in the first two years are unweighted; from age 2–4 are weighted by 0.5; age 5–11 by 0.2; and age 12–15 by 0.1.
**Repeats**	1	1.37	Four or more consecutive Dynamic exposure events.
	2	1.30	At least two instances of two consecutive years of Dynamic exposure events.
	3	1.77	At least two consecutive years of both Loss and SES exposure events.
**Order**	1	5.68	First Dynamic exposure event precedes the first Loss exposure event, with at least one occurrence of each.
	2	1.98	First two Dynamic exposure events precede the first Loss exposure event, with at least one occurrence of each.
	3	1.80	Similar to Order 1, but the effect is mitigated if SES exposure events occur.
**Timing**	1	3.10	Loss and SES exposure events occur in the same year at least once.
	2	1.11	Loss and SES exposure events occur within one year of each other at least twice.
	3	2.50	Loss and SES exposure events occur within four years of each other at least twice.

Each Life Course Pattern (LCP) is described alongside its definition and the percentage of positive outcomes. The increasing numbers in the name indicate increased complexity of the pattern

For each LCP, three separate outputs were generated that embodied the pattern, varying in complexity to evaluate model performance across increasingly challenging scenarios with the group name reflecting this. To ensure that the simulated outcomes embodied the challenges found in healthcare studies, we maintained a class imbalance similar to that of the DANLIFE cohort, with approximately 2.5% of outcomes classified as positive [39]. The rules used to generate the outcomes are deterministic and therefore, noise was added to the train set to better imitate realistic scenarios. Specifically, ten percent of positive outcomes were randomly switched to negative outcomes, while an equal number of individuals originally classified with negative outcomes were reclassified as positive. The test set remained unaltered, meaning that models that have successfully learnt the underlying pattern can theoretically correctly diagnose all individuals and achieve perfect performance.

Our approach adopts a supervised learning perspective, focusing on modelling the functional relationship between the input data $X$ and the target variable $y$. The objective is to learn a mapping function $f$ such that $y = f(X) + \epsilon$, where $\epsilon$ denotes the noise or error term. It is important to note that, by design, the precise nature of the input data $X$ has limited impact on the model performance and it is instead solely the relationship between the input data and disease outcome that drives the results. In this way, our results are applicable to many diverse healthcare datasets and contexts.

### Model architectures

We compared the predictive performance of six models on each of the multiple simulated outcomes: a baseline logistic regression (LR) model, a tree-based ML model (Extreme Gradient Boosting [XGBoost] [37]), and four DL architectures tailored for time series analysis. The DL models included: the Multivariate Long Short-Term Memory Fully Convolutional Networks (MLSTM-FCN [47]), which integrates LSTM networks for sequential learning with fully convolutional networks for capturing spatial patterns; ResNet [47], a deep residual network designed to address vanishing gradient issues while learning hierarchical representations; InceptionTime [48], a convolutional neural network optimised for multivariate time-series data utilising inception modules to capture features at various scales; and LSTMAttention [49], which employs an attention mechanism to emphasise long-range dependencies in sequential data. Each model architecture is explained in detail in the supplementary material.

To handle class imbalances effectively, various strategies were implemented to enhance model performance while minimising the risk of the model prioritising the larger negative class. The LR model employed class balancing, whereas the XGBoost model adjusted class weights in accordance with their distribution in the loss function. For the DL models, we adopted the focal loss function to prioritise the learning of harder-to-classify individuals, assigning greater weight to the minority class. The model weights were trained from scratch. Further details regarding the model architectures and fitting procedures can be found in the supplementary materials, with Tables S2 and S3 giving the hyperparameters optimised for the XGBoost and DL models, respectively.

### Model fitting & hyperparameter optimisation

Hyperparameter optimisation was conducted using the optuna package, to allow for fine-tuning of parameters such as learning rate, batch size, weight decay, and model-specific parameters (Tables S2 and S3 in the supplementary materials contain the parameters that were optimised as well as their ranges for XGBoost and the DL models, respectively). To ensure robust evaluation, we employ stratified $k$-fold cross-validation (with $k = 2$), maintaining class balance within each fold. For the ML models, the Tree-structured Parzen Estimator (TPE) sampler was employed to efficiently explore the hyperparameter space. Unpromising trials were pruned at each epoch, allowing computational resources to be focused on the most promising configurations. An early stopping mechanism was implemented to mitigate overfitting, with training halted based on validation loss improvement over a specified number of epochs, preserving the best-performing model weights. The dataset was divided into training (80%) and test (20%) sets, with each model trained for a maximum of 50 epochs.

Once hyperparameters were selected to maximise the average precision score (AP - an approximation of the area under the precision-recall curve [AUPRC]), final training was conducted using 90% of the train set, with the remaining data reserved for early stopping. Model performance was assessed using various metrics, including F1-score, area under the receiver operating characteristic curve (AUROC), and AUPRC [50, 51]. The AUPRC is particularly relevant for imbalanced datasets, as it captures the model’s ability to distinguish between the positive and negative classes [52]. Additionally, the Brier score was employed to evaluate calibration quality, providing insight into the degree of over- and under-prediction, with lower scores being preferable. A well-calibrated model should have predicted probabilities that are a true reflection of the likelihood of an event occurring, and a poorly calibrated model can be harmful to decision making [53].

### Experimental setup

The performance of LR, XGBoost, and multiple DL models (InceptionTime, LSTMAttention, MLSTM-FCN, and ResNet) was evaluated across the four LCPs: Period, Repeats, Order, and Timing (Table 1). Each LCP represents a different type of temporal pattern, testing the models’ abilities to predict binary outcomes under challenging conditions, particularly given the high class imbalance (only 2.5% positive outcomes) and the presence of 10% noise. Each model’s performance is described using F1 scores, AUPRC, AUROC, and Brier scores to assess model discrimination, precision-recall trade-offs, and calibration.

The second part of the analysis examines explainability using the SHAP methodology which quantifies the relative influence of each exposure on the model’s predictions, they are individual-level and have temporal specificity, which means it is possible to not only examine which exposures are important but also at which points in time they exert the strongest influence. The individual-level SHAP values are first studied, followed by population-level marginal SHAP beeswarm plots to investigate how closely the SHAP values align with the simulated causal trends.

## Results

### Comparison of model performance for different LCPs

**Fig. 1:**
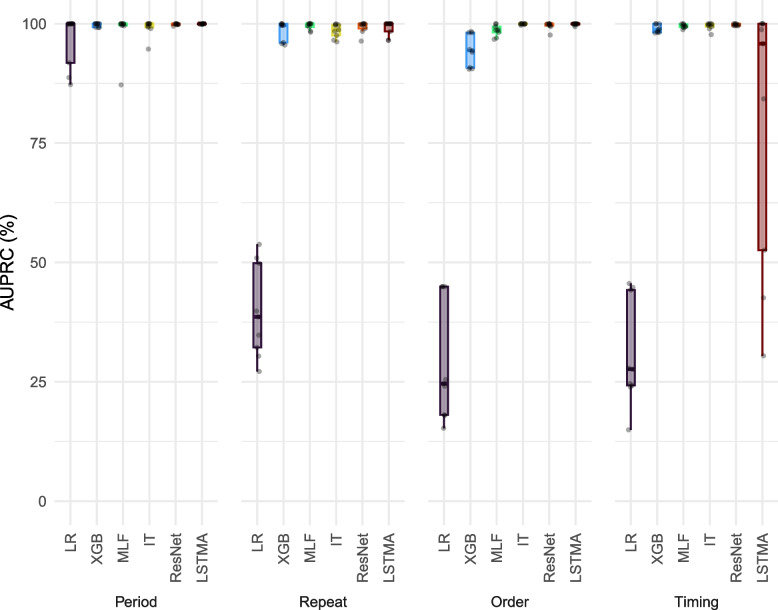
Predictive performance of models for each of the four LCPs (Period, Repeats, Order and Timing). Model performance, measured by AUPRC scores, shown for each of the three patterns that fall within each LCP. All the AUPRC scores that resulted in each of the three different seeds used to control model training and hyperparameter optimisation are shown. Boxes show the interquartile range, midpoints depict the median values and whiskers show the minimum and maximum values excluding outliers. LR = logistic regression, XGB = XGBoost, MLF = MLSTM-FCN, IT = InceptionTime, LSTMA = LSTMAttention

Figure 1 shows the model performance of the six models within each LCP measured by the AUPRC score, with higher values indicating better predictive performance. Firstly, the figure shows that LR demonstrates a sharp decline in AUPRC as the LCP pattern complexity increases and incorporates relative temporal and inter-variable dependencies, reflecting its limited capacity to handle non-linear and temporal patterns. Similarly, XGB also exhibits a drop in performance with increasing complexity, albeit less steep than LR. Both of these models perform comparably to the DL models for the Period LCP but fall behind once the LCPs become dynamic. Secondly, across the four LCPs, there is no single DL model that consistently outperforms the rest with DL performance exhibiting variability depending on the underlying causal structure. Another point of note is that the LSTMAttention model performs poorly for the Timing LCP overall, demonstrating vulnerability despite its excellent performance on the other LCPs. The model performance measured by the AUC score is shown in Figure S6.

The different evaluation metric values averaged across the three random seeds are displayed in Table 2. Further threshold-dependent evaluation metric values are provided in the supplementary materials (Table S4). Higher scores reflect greater discriminative performance for all metrics except for the Brier score where lower values indicate better model calibration. There are several instances where there is a big discrepancy between model performance when measured by the AUROC score compared to the AUPRC and F1 scores. For example, LR frequently achieved high AUROC values when the associated AUPRC scores were very low, for example, the AUPRC scores for Order3 and Timing1 were 17.12% and 26.59%, respectively, whilst the AUROC exceeded 90%.

**Table 2:** Relative model performance within four LCPs; period, repeats, order, and timing

	LR				XGBoost				MLSTM-FCN				InceptionTime				ResNet				LSTMAttention			
	F1	PRC	AUC	Brier	F1	PRC	AUC	Brier	F1	PRC	AUC	Brier	F1	PRC	AUC	Brier	F1	PRC	AUC	Brier	F1	PRC	AUC	Brier
Period																								
1	**100.00**	**100.00**	**100.00**	0.07	99.92	**100.00**	**100.00**	**0.03**	**100.00**	**100.00**	**100.00**	4.10	99.92	99.99	**100.00**	0.25	99.89	99.94	99.95	0.12	**100.00**	**100.00**	**100.00**	1.05
2	99.82	99.96	**100.00**	0.25	99.93	**100.00**	**100.00**	0.02	99.67	99.93	**100.00**	0.03	99.71	99.84	99.92	0.06	99.82	99.95	**100.00**	0.09	**100.00**	**100.00**	**100.00**	**0.01**
3	81.09	89.26	99.69	0.50	97.15	99.22	99.97	0.12	91.91	95.47	99.88	1.17	95.96	97.65	99.97	0.21	**98.61**	99.73	**99.99**	**0.06**	98.47	**99.89**	**100.00**	**0.06**
Repeat																								
1	50.42	51.52	98.23	0.92	99.45	99.98	**100.00**	0.04	99.82	99.99	**100.00**	0.06	99.33	99.84	99.99	0.06	99.51	99.95	**100.00**	0.05	**99.94**	**100.00**	**100.00**	**0.01**
2	39.38	30.78	95.91	1.08	**98.28**	**99.68**	**100.00**	**0.05**	97.84	98.67	99.95	0.14	96.96	97.06	99.92	1.37	97.88	98.45	99.39	0.10	96.37	97.15	99.97	0.14
3	42.29	36.87	97.53	1.40	90.07	95.81	99.92	0.33	99.67	99.87	99.99	0.05	96.88	98.68	99.97	3.93	98.28	99.38	99.99	0.10	**100.00**	**100.00**	**100.00**	**0.02**
Order																								
1	57.48	44.93	95.67	4.20	97.70	98.20	99.93	0.37	99.04	99.39	99.63	2.64	**99.80**	**99.99**	**100.00**	2.48	99.76	99.95	**100.00**	**0.07**	98.89	99.80	99.99	0.20
2	34.57	24.72	95.94	1.77	86.39	90.63	99.79	0.47	95.48	97.50	99.44	0.20	98.82	99.84	99.99	0.05	97.46	98.97	99.82	0.16	**99.37**	**99.96**	**100.00**	**0.04**
3	27.24	17.12	93.75	1.70	91.81	94.34	99.88	0.35	98.60	98.89	99.37	0.08	99.58	**99.98**	**100.00**	0.02	99.35	99.79	99.92	0.03	**99.86**	99.90	99.99	**0.01**
Timing																								
1	36.49	26.59	93.47	2.67	99.73	99.99	**100.00**	0.06	99.22	99.95	**100.00**	0.83	99.89	99.99	**100.00**	0.07	**100.00**	**100.00**	**100.00**	0.43	**100.00**	**100.00**	**100.00**	**0.01**
2	28.26	21.15	95.76	0.99	95.03	98.71	99.98	0.16	97.01	99.40	**99.99**	**0.06**	95.77	98.68	99.95	0.42	**97.98**	**99.70**	**99.99**	0.08	77.01	75.01	99.47	1.81
3	54.40	44.87	98.17	1.83	93.81	98.15	99.92	0.31	97.86	99.21	99.84	0.16	**99.24**	**99.80**	99.97	**0.04**	98.97	99.67	**99.99**	0.06	65.41	59.82	97.54	25.05

Each row gives information about a rule including the LCP it falls into, an identifying number, the proportion of the resulting outcome that is positive and its definition. If the rule is met, then the outcome is positive. For each model the F1, AUROC and AUPRC values as percentages are provided and the highest performing model for each dataset is highlighted in bold font. 10 percent noise in the data. LR = logistic regression. Note F1 scores depend on the chosen threshold which in this case was selected to maximise the F1 score. All other metrics are independent of the threshold

For the Period LCPs, all models have exceptionally good performance for the simpler patterns, with AUROC and AUPRC scores approaching 100%. Notably, LSTMAttention showed perfect performance for rules Period1 and Period2 which typify critical and sensitive periods, with all metrics, including Brier scores, indicating a robust fit to the data. All other models, including LR and XGBoost, also excelled in discriminative performance and had Brier scores indicating good calibration, although MLSTM-FCN showed a slightly elevated Brier score of 4.10 for rule Period1. As the complexity of Period patterns increased from Period1 to Period3, differences between models became evident. LR, although it performed comparably for the other rules, struggled particularly with the Period3 rule which incorporates different weights for the impact of an exposure according to the time when it occurred, achieving an AUPRC of only 89.26%. Moreover, the AUPRC values for the MLSTM-FCN (95.47%) and InceptionTime (97.65%) were lower than XGBoost (99.22%) as well as all other DL models (99.73–99.89%).

The Repeats patterns show substantial disparities in model performance. LR consistently underperformed compared to the other models, with AUPRC values consistently below 52% and as low as 30.78% for rule Repeats2. In contrast, LSTMAttention, MLSTM-FCN and ResNet performed exceptionally well. LSTMAttention reached an AUPRC of 100% for rule Repeats1, reflecting perfect precision-recall balance, and maintained an AUROC of 100% across all rules. MLSTM-FCN and ResNet also demonstrated high predictive performance, with AUPRC scores above 98% and Brier scores indicating strong calibration. InceptionTime followed closely behind in terms of predictive performance but the associated Brier scores were markedly higher than the other models with values as high as 3.93% for Repeats3. In contrast, the performance of XGBoost varied, achieving strong metrics for simpler rules (rule Repeats1, AUPRC 99.98%) but experiencing greater declines in performance for complex patterns, giving F1 scores as low as 90.07% as seen in rule Repeats3.

Order patterns, which involve the sequential ordering of events, further highlighted differences in model performance. InceptionTime stood out with near-perfect scores, particularly for rule Order3, where it had an AUPRC of 99.98% and a very low Brier score of 0.02. LSTMAttention similarly performed well, with an AUROC and AUPRC of 100% for rule Order1. On the other hand, LR exhibited significant weaknesses, with AUPRC values falling to 24.72% and 17.12% for rules Order2 and Order3, respectively, despite maintaining AUROC scores above 90%. Higher Brier scores accompanied the poor predictive performance with values as high as 4.2% for Order1. ResNet and MLSTM-FCN scored consistently high metrics with AUPRC scores above 97%, while XGBoost displayed reduced performance for more complex order rules, such as rule Order2 (AUPRC 90.63% and F1 score 86.39%), particularly in precision-recall metrics.

The Timing patterns were also challenging for LR, with rule Timing1 showing particularly poor outcomes, as evidenced by an AUPRC of only 26.59% and a Brier score of 2.67, indicating poor calibration and difficulty in modelling the timing of events. Generally, DL models outperformed LR significantly. However, LSTMAttention, while having the highest performance for the other LCPs, struggled with rules Timing2 and Timing3, achieving AUPRC scores of 75.01% and 59.82%, respectively, despite AUROC scores greater than 97%. ResNet maintained high performance with all AUPRC values greater than 99.67% and low Brier scores, suggesting excellent calibration. InceptionTime and MLSTM-FCN both performed very well, followed by XGBoost which had slightly lower AUPRC scores but markedly lower F1 scores that dropped to 93.81% for the rule Timing3.

### Explainability

**Fig. 2:**
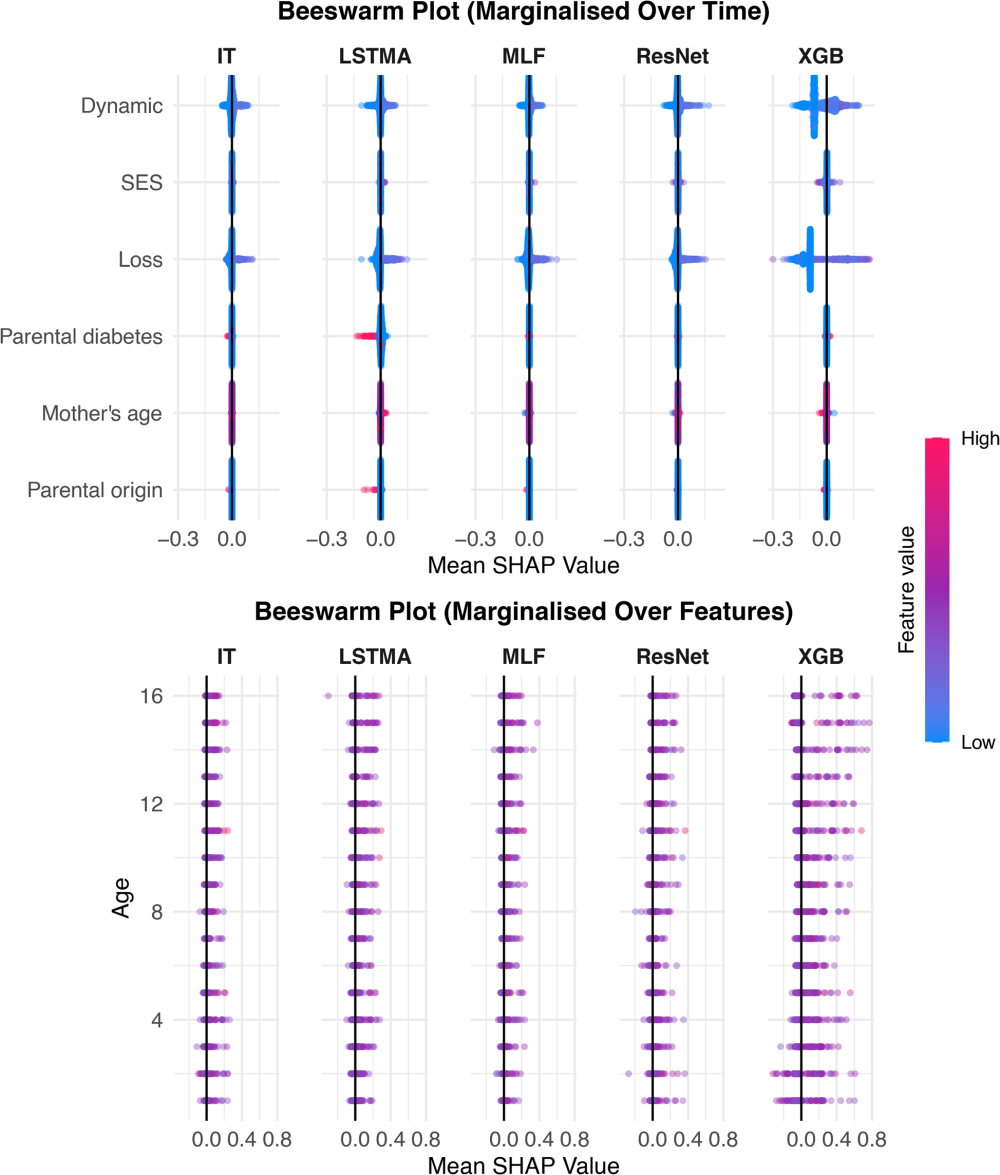
SHAP Beeswarm Plots for LCP Order1. The top panel illustrates SHAP values marginalised over time for each feature, grouped by model, with features ordered by mean absolute SHAP values. The bottom panel visualises SHAP values marginalised over features for each age, also grouped by model. Feature values are colour-coded, ranging from low (“Low”) to high (“High”). LR = logistic regression, XGB = XGBoost, MLF = MLSTM-FCN, IT = InceptionTime, LSTMA = LSTMAttention

Figure 2 shows population-level beeswarm plots for the LCP Order3 marginalised over time and the features, separately. This pattern generates a positive outcome if at least one Dynamic and Loss exposure event occur with the first Dynamic exposure event preceding the first Loss exposure event. The beeswarm plot marginalised over time shows that the majority of the models identify Dynamic and Loss variables as being the most influential on model predictions. All models show similar patterns with lower values of these features, i.e. non-occurrences, being more likely to have negative SHAP values meaning that positive predictions depend on the occurrence of these exposures. The pattern is most pronounced for XGBoost, followed by ResNet and MLSTM-FCN. Notably, InceptionTime shows very limited variation in SHAP values, unlike the other models, and the pattern of lower SHAP values for lower feature values is very slight. These discrepancies show that the models do not agree on the most influential factors. Additionally, the SHAP values for individuals whose parents have diabetes (high feature value) appear to negatively influence model predictions for LSTMAttention alone. The second beeswarm plot, showing SHAP values marginalised over the features, has no clear discernable trend. It is notable that, again, XGBoost has the greatest spread in the magnitude of SHAP values, however it is not possible to discern any temporal dependencies. Therefore, whilst it is possible to use these plots to gain insight into the influence of the occurrence of multiple exposure events, these population-level plots alone do not reflect dynamic causal relationships.

In some very simplified scenarios, it is possible to correctly guess the causal mechanism from the population-level beeswarm plots alone. Figure S7 in the supplementary materials, shows the marginalised beeswarm SHAP plots for the Period3 pattern; a simple linear pattern that assigns greater weights to earlier Loss exposures in calculating the likelihood of a positive outcome. The beeswarm plot marginalised over time clearly picks out the Loss feature as the only influential feature and the lower SHAP values associated with lower feature values suggests that the greater number of Loss events increases the likelihood of a positive outcome. The beeswarm SHAP plot marginalised over time links greater feature values at younger ages with higher SHAP values, and the magnitude of the SHAP values gets lower with age. Due to the linear nature of this LCP, it is possible to piece these two pieces of information together to link the occurrence of more Loss exposures at younger ages with the outcome which is consistent with the causal pathways in the generated data. However, the marginalised SHAP beeswarm plots for the non-linear patterns are not as easily interpretable.

**Fig. 3:**
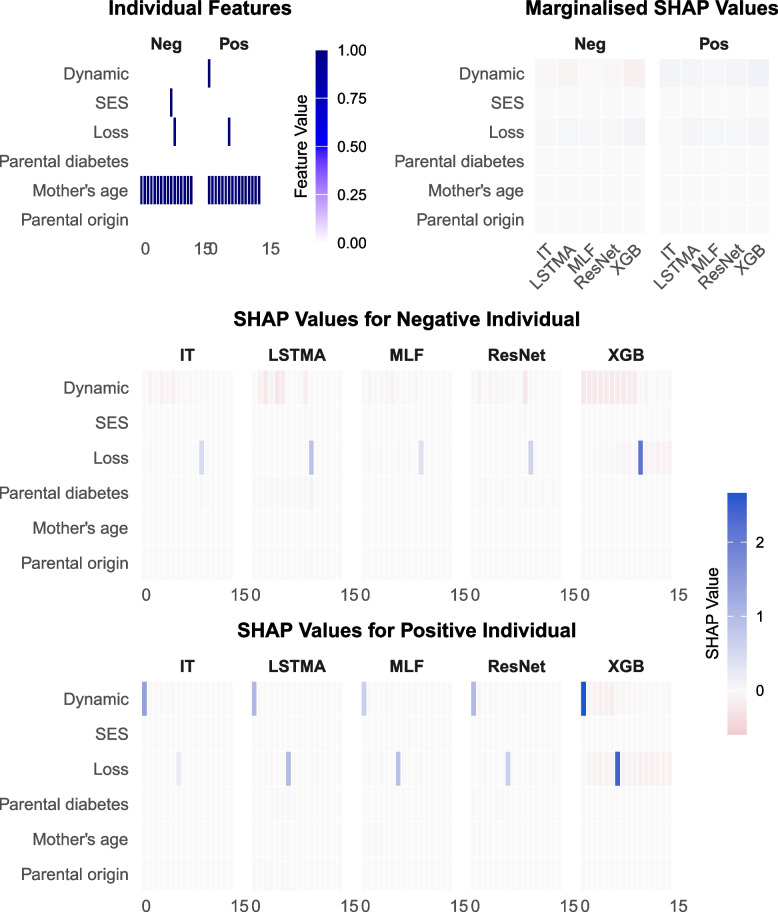
SHAP Comparison for Individuals for LCP Order1. This figure compares SHAP values for two individuals, one positive for the outcome of interest and one negative. The top row includes individual feature heatmaps and SHAP values marginalised over features. The bottom two rows depict the individual-level SHAP values marginalised over time for each individual split by model. SHAP values are shown on a consistent scale across all plots, with colour gradients indicating magnitude and direction (blue to red). LR = logistic regression, XGB = XGBoost, MLF = MLSTM-FCN, IT = InceptionTime, LSTMA = LSTMAttention

Figure 3 shows the individual-level data and related SHAP values for two individuals (one positive and one negative). The SHAP values for the positive individual have large positive values associated with when the Dynamic and Loss exposure events occur. This shows that these two events have the greatest influence on the positive model prediction. It is also possible to see that the non-occurrence of Dynamic and Loss events, particularly early Dynamic events and late Loss events, have small negative impact on the model prediction. This is most noticeable with XGBoost, which also has the SHAP values of the highest magnitude, followed by ResNet. Similarly, the negative individual also has the loss exposure event increasing the likelihood of a positive outcome. However, each model has multiple negative SHAP values related to the non-occurrence of any Dynamic events prior to the Loss exposure event, resulting in the correct negative model prediction. These patterns directly align with the causal pathways in the simulated data. Finally, notice that the marginal plots obscure the temporal relationships as the SHAP values all have small magnitudes.

Using individual-level SHAP values to identify the temporal relationships is also possible for other LCPs. For instance, the Timing1 LCP also has causal patterns that are easy to discern from individuals. Figure S8 in the supplementary material shows that the negative individual has positive SHAP values associated with the occurrence of the single Loss exposure event indicating that this event increases the likelihood of positive classification. However, this is offset by an equal or greater negative SHAP value related to the non-occurrence of a SES event in the same year. The magnitude of the SHAP values vary greatly between the different models. The positive individual has large positive SHAP values when the Loss and SES events co-occur. As in the negative case, some models, such as XGBoost, assign positive SHAP values to the additional occurrences of SES and Loss exposure events and corresponding negative values to the lack of the other. Moreover, several of the DL models assign small SHAP values to the other exposure events in ways that do not relate to the underlying causal relationships.

There are other LCPs and individuals for which is is significantly harder to discern the causal relationships from the individual-level SHAP values, where the SHAP values bare little resemblance to the underlying simulated mechanisms. Figure 3 in the supplementary material shows the SHAP values and individual-level data for the Order3 LCP, where a positive outcome is generated if there is no SES exposure event and a Dynamic exposure occurs prior to a Loss exposure. Some models, primarily XGBoost and ResNet, have negative SHAP values aligning with the SES exposure event, correctly identifying its existence as acting against a positive outcome. However, this SHAP value for many models is approaching zero and incorrectly indicating that this exposure has little influence on model predictions. For the positive individual, the SHAP values related to the SES feature are all slightly positive at the non-occurrence of any exposure events, showing that this is important in generating a positive outcome. Moreover, the positive SHAP values associated with the single Loss exposure event suggest that this event is increasing the likelihood of the positive outcome, although the magnitude exhibits substantial variation between models. However, the Dynamic exposure events are assigned a variety of different SHAP values by each of the models. InceptionTime associates all Dynamic events with negative SHAP values, indicating that this event occurring negatively influencing model predictions, when the opposite is true. XGBoost has both positive and negative SHAP values for the different Dynamic events which is also contrary to the simulated pattern, as only the first Dynamic exposure event is in the LCP mechanism. None of the models correctly identify the significance of this feature in the underlying causal relationship.

## Discussion

This study provides the first systematic evaluation of DL models and XAI methods for life course epidemiology. While DL is widely used in healthcare research, its application to life course data remains underexplored. Our findings demonstrate that DL models outperform traditional epidemiological approaches, such as LR and XGBoost, in identifying LCPs, particularly those involving complex temporal and intervariable dependencies between the exposures and the outcome. XGBoost performed well for simpler LCPs, however it was outperformed by DL models as pattern complexity increased, supporting evidence that DL models are better suited for dynamic, high-dimensional data [54–56], despite XGBoost’s advantages in tabular datasets [57]. One key finding of this study is the absence of a universal best-performing DL model, reinforcing the importance of selecting models according to the specific datasets [16]. These findings underscore the importance of context-aware model selection to maximise predictive performance in life course research.

The findings of this study build on existing research demonstrating the strengths of DL for longitudinal data analysis in healthcare [36, 58, 59]. However, most prior research has focused on EHRs rather than life course data, which presents additional challenges including sparse exposure histories and long-term dependencies. While DL models appear to be well-suited to handling sparsity [35, 36], empirical validation has been limited. Our results confirm that DL models can handle these complexities better than traditional methods.

### Explainability & causality

Despite their predictive advantages, DL models present interpretability challenges, particularly in epidemiological contexts where causal inference is a key objective. This study evaluated SHAP, a widely used post-hoc XAI method, to assess how well SHAP explanations align with causal patterns in life course exposures. While SHAP performed well for simple LCPs, it became increasingly misaligned as the simulated exposure-disease relationships became more complex, often identifying variables inconsistent with the true simulated causal pathways. These findings support broader concerns that post-hoc XAI methods struggle with high-dimensional, temporally structured data [24, 25, 30, 31]. Recent research has raised concerns about the sensitivity of SHAP to correlated variables and confounding [33, 34], with studies observing discrepancies between SHAP explanations and true causal mechanisms in biomedical datasets [28]. Our results reinforce these concerns, highlighting the need for alternative XAI techniques that better align with epidemiological principles.

Several alternative XAI methods could have been considered for this study [29, 60]; however, each has limitations when applied to longitudinal health data. Gradient-based methods such as Integrated Gradients (IG), Deep Learning Important Features (DeepLIFT), and Layer-Wise Relevance Propagation (LRP) provide feature importance scores via backpropagation [61, 62] but struggle with capturing feature interactions and generalising across architectures [63, 64]. Additionally, these methods lack temporal modelling, making them less suitable for life course analysis. Model-agnostic approaches, such as Local Interpretable Model-Agnostic Explanations (LIME) and Anchors, approximate model behaviour with simpler interpretable models [26, 65, 66], but their sensitivity to parameter choices and instability in high-dimensional datasets limit their reliability. Attention-based techniques can highlight influential time points in sequential data, but may introduce biases from patterns in the training data [22].

Shapley Interaction Quantification (SHAPIQ) is a recently developed approach that offers a promising alternative by refining feature importance estimates in the presence of correlated exposures and evolving dependencies over time, making it particularly valuable for longitudinal epidemiology [67]. Emerging causal-aware methods, such as CausalSHAP and counterfactual explanations, aim to provide causal insights but often require predefined causal structures, which may not be feasible in observational health studies [68, 69]. In contrast, SHAPIQ offers a flexible alternative that does not require an explicit causal graph. Our findings suggest that SHAPIQ could improve interpretability in complex epidemiological models, particularly in situations involving correlated exposures, temporal dependencies, and confounding.

As DL and XAI methods become increasingly integrated into healthcare decision-making and policy, careful consideration of their limitations is essential. Misinterpreting model outputs-such as equating predictive markers with causal significance-can lead to poorly designed interventions that risk exacerbating health inequalities or introducing unintended harms. Clinicians and policymakers must recognise that XAI tools like SHAP do not inherently provide causal explanations. Our findings underscore the need for rigorous validation, transparency, and appropriate methodological safeguards when applying these models in public health, ensuring that policy decisions are not based on incorrect assumptions about causality

### Study limitations and future research

Several limitations of this study should be acknowledged. First, while our use of simulated datasets enabled controlled evaluations of model performance and interpretability, it also has inherent limitations. Simulated data may not fully capture the complexities of real-world life course data, which are often subject to measurement error, unmeasured confounding, and selection bias. As a result, the relative simplicity of the simulated data may have contributed to the somewhat elevated model performance observed in this study. Future research should extend our findings by evaluating DL models on empirical life course datasets to assess their performance under real-world conditions. Second, although we focused on SHAP due to its widespread adoption, other XAI methods warrant further investigation. Epidemiological applications frequently require causal insights as well as insights into variables influential on predictive performance. Existing XAI tools primarily identify predictive markers rather than causal relationships, limiting their applicability for causal inference in life course research. Therefore, an approach such as SHAPIQ or CausalSHAP may be suitable for this area, with the potential to improve interpretability by aligning feature attributions with underlying causal structures [70]. Future research should focus on developing or adapting XAI methods for longitudinal data and life course research, by integrating causal inference principles and explicitly accounting for both temporal dependencies and complex feature interactions. Third, the high computational demands of DL models, particularly for hyperparameter tuning and training, present practical constraints for large-scale epidemiological studies. These computational requirements may limit the feasibility of implementing DL methods in resource-constrained settings and any changes made to simplify the model training pipeline may lead to reduced performance and utility.

### Conclusions

This study has several important implications for both life course epidemiology and healthcare systems as a whole. First, with healthcare systems increasingly digitising and register-based datasets expanding, there is an unprecedented opportunity to leverage DL models to uncover key risk factors for a wide range of health conditions. These models can facilitate a deeper understanding of how the timing, intensity, and cumulative impact of exposures experienced throughout the life course influence long-term health outcomes. By identifying and analysing these complex patterns, DL methods have the potential to uncover novel relationships that might otherwise remain hidden, enabling more precise insights into disease mechanisms. This capability is particularly important for advancing precision public health, where tailored interventions can be designed based on a more nuanced understanding of how different risk factors interact over time. In this regard, our findings suggest that when life course patterns are present in the data, DL models show the most promise in unravelling these intricate relationships. By integrating time-sensitive and cumulative risk factors, DL could open new pathways for designing interventions and clinical prediction models that are more responsive to individual health trajectories and more sensitive to the timing of exposures. This offers the potential to significantly improve public health interventions, enabling them to be more context-specific and effective.

This study demonstrates the potential of DL and XAI to advance life course epidemiology. Our findings suggest that while DL models can capture complex exposure-disease relationships, model selection should be context-dependent. Moreover, we caution against over-reliance on post-hoc XAI methods for deriving causal insights and emphasise the need for continued innovation in XAI tools that consider causal principles. By addressing these challenges, DL and XAI methods have the potential to transform life course epidemiology, offering novel insights into disease risk trajectories and informing more effective public health interventions.

## Supplementary Information

Supplementary Material 1.

## Data Availability

Data is provided within the manuscript or supplementary information files.
