# Supplementary material for: Exploring the potential and limitations of deep learning and explainable AI for longitudinal life course analysis
Source: BMC Public Health. 2025 Apr 24;25:1520. doi: 10.1186/s12889-025-22705-4 (PMC12020094; doi:10.1186/s12889-025-22705-4)
Supplement: Supplementary file 1 — Supplementary Material 1. [file 12889_2025_22705_MOESM1_ESM.pdf]

Supplementary

Deep Learning Models

LSTMAttention

Long short-term memory (LSTM) models incorporating attention mechanisms (LSTMAttention) have been developed to enhance the ability to capture sequential dependencies in time-series data by assigning varying levels of importance to different time points[1]. The attention mechanism, originally introduced in the Transformer model for natural language processing (NLP) tasks by Vaswani et al.[2], has been successfully adapted for temporal data modelling, allowing for improved efficiency in sequence learning.

The LSTMAttention model is designed with stacked layers that extract both short- and long-range temporal dependencies. A key feature is its self-attention mechanism, which dynamically adjusts the relevance of time steps within the sequence. This enables the model to prioritise important time points without processing data sequentially, as required by conventional recurrent neural networks (RNNs). Additionally, multi-head attention mechanisms allow the model to learn various data relationships in parallel, increasing both computational efficiency and the ability to model complex patterns in sequential data. The architecture consists of several stacked layers, each integrating self-attention and feed-forward neural networks. Positional encodings help the model maintain temporal context, while residual connections enhance stability and training efficiency.

In the adaptation of the Transformer model for time-series applications, the model is composed only of the encoder component of the original model. The encoder is formed of six identical blocks, each of which contains a multi-head self-attention sub-layer followed by a feed-forward network. Using self-attention layers enables faster computation and improves the model's ability to learn long-range temporal dependencies[1]. The feed-forward network takes the form of a pointwise, fully connected layer with each of the six blocks having its own trainable weights and biases. Around each of the sub-layers is a residual connection that allows that layer to be skipped if its inclusion in the model does not improve training. Between each sub-layer there is a batch normalisation layer. Following the six model blocks, there is a linear transformation then a softmax activation function to output the class predictions. Prior to being processed by the model, input data is projected into a vector space, and positional encodings are incorporated to retain sequence information.

Multivariate Long Short Term Memory Fully Convolutional Networks

Multivariate Long Short-Term Memory Fully Convolutional Networks (MLSTM-FCNs) are an advanced deep learning framework designed for multivariate time series analysis. This architecture integrates two complementary neural network models: Fully Convolutional Networks (FCNs), known for their proficiency in spatial feature extraction, and Long Short-Term Memory (LSTM) networks, which are adept at capturing long-range dependencies in sequential data[3]. By combining these components, MLSTM-FCNs can process complex temporal patterns while maintaining a structured understanding of variable interactions across time[4]. These models have been successfully applied across various domains, including earthquake detection and healthcare, particularly in arrhythmia classification[5,6].

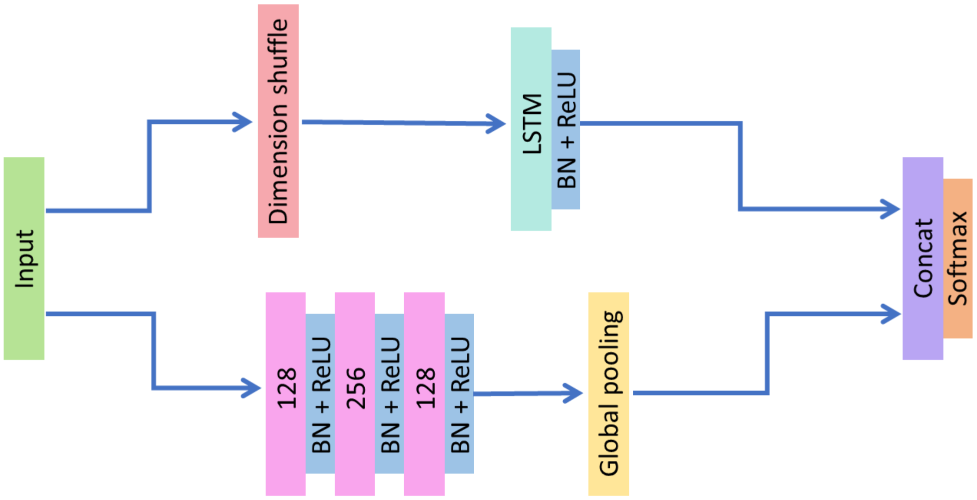

Figure S1. LSTM-FCN model structure. Adapted from Karim et al.[4].

The FCN module is formed of three convolutional layers with progressively smaller kernel sizes (to extract both fine-grained and high-level temporal features. Convolutional filters slide across the input sequence to capture local patterns and create feature maps. Between each layer is a layer of batch normalisation and a ReLU activation function. Then the output of the FCN is put through a global average pooling layer, which reduces dimensionality while preserving critical information. In parallel, the LSTM component processes the input data by first passing it through a dimension shuffle layer, which transposes the temporal dimension (Figure S1). This transformation ensures that the LSTM receives a multivariate time-series representation with a single time step, significantly improving training speed. The outputs from both the FCN and LSTM branches are concatenated, enabling the model to leverage both sequential and spatial information. A final softmax layer provides classification probabilities.

Residual Network

Residual Network (ResNet) is a deep neural network architecture that is widely used in time-series analysis. Originally introduced by He et al. for image recognition, ResNet has since been adapted to various machine learning domains, including time-series classification, signal processing, and speech recognition[7–10]. The adaptability of ResNet models makes them particularly valuable for MTS data, where dependencies across different variables and time steps must be effectively modelled. Their defining feature is the introduction of residual connections—also called shortcut or skip connections—which mitigate the vanishing gradient problem and enable deeper networks to be trained effectively. In essence, if certain layers do not contribute meaningfully to the learning process, the residual connections allow the network to return to simpler representations, similar to an ensemble of shallower models within the deep structure.

This ResNet model for time-series classification consists of multiple stacked residual blocks, each containing several convolutional layers[3] (Figure S2). ResNet is composed of three blocks, each of which consists of three convolutional layers that apply one-dimensional filters to capture temporal dependencies at different scales. Between each of the layers batch

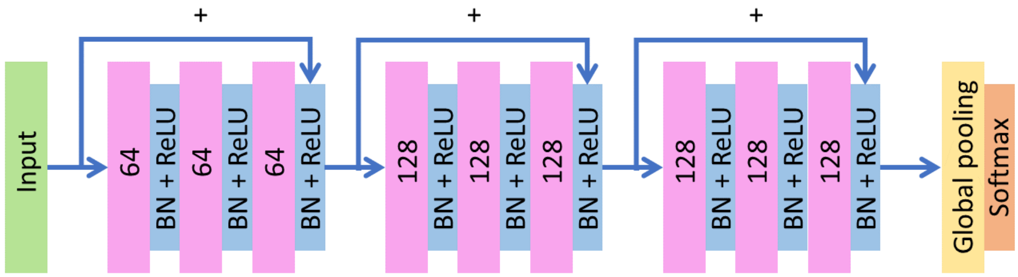

Figure S2. ResNet model structure. Adapted from Wang et al.[3].

normalisation and a GeLU activation function are applied to stabilise training. Across each block lies a residual connection in which the original input of the block is added to the transformed output before passing through another activation function. In the first of the three blocks, each convolutional layer has 64 one-dimensional filters with kernel length of 7, 5 and 3 for the first, second and third layers, respectively. Using different filter lengths enables temporal information to be extracted at differing degrees of granularity. The remaining two blocks are identical to the first except that each convolutional layer has 128 filters instead of 64. After the three blocks, there is a global average pooling layer which aggregates information across all time steps. Finally there is a softmax layer to obtain the final classification labels.

InceptionTime

InceptionTime is a DL architecture designed for time-series classification (TSC) tasks by He et al.[7].It is an adaptation of the Inception model which was designed for image recognition[11]. InceptionTime extends the concept to sequential data by incorporating multiple parallel convolutions. This design allows the model to efficiently capture both short-term fluctuations and long-range dependencies within MTS data[12]. The InceptionTime model has repeatedly outperformed other CNN-based DL architectures in several applications including detecting cardiovascular abnormalities and classifying ECGs and classifying brain activity[13–15]. The architecture has consistently outperformed conventional CNN-based models across various TSC benchmarks, reinforcing its status as a state-of-the-art framework for sequential data analysis.

The structure is composed of six "inception modules" that are arranged into two identical blocks that each contain three modules (Figure S3). Each block has a residual connection across the top to improve gradient flow and improve model stability during training [7]. After the two blocks, there is a global average pooling layer followed by a fully connected softmax layer to predict class labels. Between each of the modules is a batch normalisation and GeLU activation function. Each inception module consists of multiple convolutional layers operating in parallel, each with a distinct kernel size. This parallel structure enables the model to extract features at different temporal granularities, ensuring both local and global patterns are learned effectively. Following the inception blocks, a global average pooling layer aggregates extracted features before passing them through a softmax layer for final classification.

The inception modules are composed of multiple parallel convolutional filters of varying lengths that are concatenated to form the output (Figure S4). Firstly, the data passes through a bottleneck layer which reduces the dimensionality of the

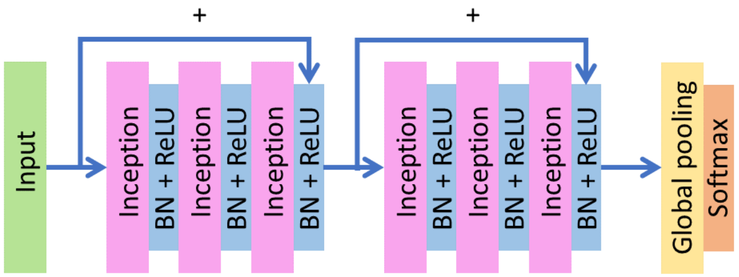

Figure S3. InceptionTime model architecture Adapted from Fawaz et al.[12].

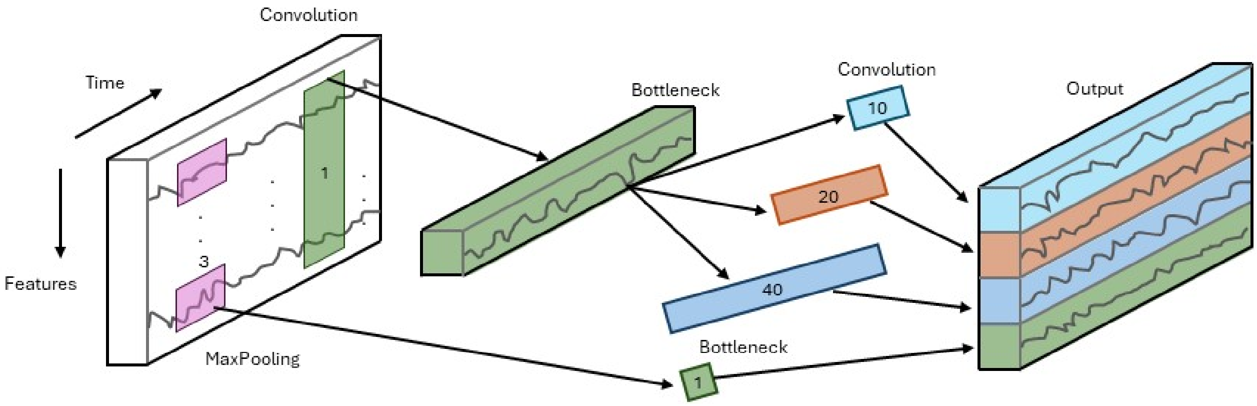

Figure S4. Inception module structure. Adapted from Fawaz et al.[12]. The input MTS is processed to form the output MTS that reflects temporal patterns in the data.

data and also computational complexity. The bottleneck layer is a 1D convolutional layer with kernel size 1 and filter length 32. Following the bottleneck layer there are three parallel convolutional layers with kernel sizes 10, 20 and 40 with filter size 32. The convolutional layers are designed to be able to pick up on short-, mid- and long- term temporal dependencies in the data, meaning that InceptionTime is appropriate for sparse temporal data[11]. Depthwise separable convolutions are used which factorise the standard convolution into a depthwise convolution followed by a pointwise convolution. There is a further additional layer that runs in parallel to the bottleneck and convolutions that consists of a max pooling layer with window size 4 then the output is passed through a bottleneck layer. The results from each convolutional layer as well as this additional layer are concatenated to produce the output of the inception module.

Simulated data

Input data

The simulated cohort data, denoted as $X \in \mathbb{R}^{N \times M \times T}$, represents 100,000 individuals followed for 16 years. This cohort size, approximately half that of the DANLIFE cohort which comprises of 207,445 individuals, allows for effective model testing, with results expected to scale to larger sizes. The synthetic data was generated to simulate the dynamics of longitudinal variables based on the structure and relationships observed in the DANLIFE dataset. The DANLIFE dataset used by Davis et al. is composed of 5 variables used as covariates, namely the age of the mother at first birth, the age of the participant's mother,

parental origin, parental diabetes and the fitted trajectory group based upon adverse childhood experiences (ACEs), all of which are stationary[16]. The relationships between the six variables were simulated using a Direct Acyclic Graph (DAG) in R with the `simcausal` package (v0.5.4)[17].

The first three variables of the simulated (parental ethnicity, parental diabetic status, and maternal age at birth) are stationary, and the definitions are adopted from Davies et al.[16]. The age of the participant's mother is not simulated in the synthetic data. Baseline variables were modelled as categorical distributions with probabilities derived from the observed distributions in the DANLIFE dataset. Parental origin (European or non-European) is randomly assigned to match the distribution in the DANLIFE cohort where 98.6% of people had parents of European origin. Maternal age is divided into three categories and is also randomly assigned to the population according to the distribution in the DANLIFE data because there was no observed correlation of maternal age with parental origin. Therefore, 4% of the simulated population had mothers aged under 20 years old at the time of their birth, 75% had mothers between the ages of 20 and 30 years and the remainder had mothers older than 30 years old. Parental diabetic status is assigned to individuals depending on their parental origin because a correlation was observed in the DANLIFE data, meaning that 10% of individuals with European parental origin and 40% of individuals with non-European parental origin had parents with diabetes.

In the DANLIFE cohort, ACEs are represented by a single categorical variable that classifies individuals into trajectory groups based on three distinct ACE categories: Loss, Dynamic, and Socioeconomic Status (SES)[18]. To better capture temporal dependencies and the relative timing of exposures, we replaced this static variable with three time series, each representing the annual count of ACE events within its respective category. To model the temporal evolution of these ACE exposures, we used zero-inflated Poisson (ZIP) regression, which accounts for overdispersion and the presence of excess zeros (i.e., individuals without ACE events in a given year). Each ACE time series was influenced by its past values as well as each individual's values of the stationary covariates, including parental origin, maternal age category, and parental diabetes status. Exploratory analysis revealed that Dynamic ACE exposures were significantly influenced by the concurrent values of both Loss and SES, while no such dependencies were found in the reverse direction. That is, an increase in Loss or SES events in a given year was associated with a higher likelihood of Dynamic events occurring in the same year, but changes in Dynamic did not significantly impact subsequent Loss or SES. We incorporated this dependency structure into the DAG to reflect the underlying causal mechanisms. The ZIP distribution was fitted to the variables in the DANLIFE cohort and the resulting coefficients were used to simulate the time-dependent variables in the simulated cohort. The coefficients of the ZIP distributions can be found in the github repository.

The DAG explicitly modelled the recursive nature of ACE exposures, where each time-dependent variable (X_t) was influenced by its prior value (X_{t-1}), stationary covariates, and, in the case of Dynamic, concurrent values of Loss and SES. To prevent unrealistic values, exposure counts were capped at predefined thresholds when computing the next time step, corresponding to the 99.5th percentile of the observed data (Loss = 1, SES = 1, Dynamic = 2). SES was further constrained to a maximum value of 3, consistent with its construction in the DANLIFE dataset. By incorporating these temporal dependencies and interactions into the simulation, our approach ensured that both the direct and cumulative effects of ACE exposures were

Table S1. Distribution Differences Between DANLIFE and Synthetic Datasets Across Time-Independent Variables. This table presents the percentages (%) of individuals within each of the different groups for the three categorical time-independent variables, comparing the DANLIFE and synthetic datasets. The population size of the DANLIFE cohort used here is 207,445 and the simulated cohort has 100,000 individuals. Variable definitions are taken from Davies et al.[16].

Parental Origin	Mother's age at time of birth (years)	Parental diabetes	DANLIFE (%)	Synthetic (%)
European	< 20	No	3.51	3.55
European	< 20	Yes	0.32	0.41
European	20–30	No	67.37	66.47
European	20–30	Yes	6.90	7.66
European	> 30	No	17.66	18.46
European	> 30	Yes	2.85	2.06
Non-European	< 20	No	0.10	0.03
Non-European	< 20	Yes	0.03	0.03
Non-European	20–30	No	0.60	0.63
Non-European	20–30	Yes	0.36	0.39
Non-European	> 30	No	0.13	0.17
Non-European	> 30	Yes	0.17	0.14

appropriately captured. This framework allowed us to examine not only the individual trajectories of Loss, Dynamic, and SES over time but also their interplay with baseline characteristics, thereby enhancing the robustness and credibility of the study.

Comparison of Synthetic and DANLIFE cohorts

Here we present statistical summaries showing how closely aligned the synthetic data is with the DANLIFE dataset. Table S1 shows the percentages of individuals that fall within each subpopulation in the two datasets according to the values of their time-independent variables (parental origin, mother's age at birth and parental diabetes). The percentages of individuals in each group are very similar in both datasets, with none of the percentages differing by more than 1 percent. Therefore, the composition of the cohorts is very similar across the stationary variables and the dependencies in the DANLIFE cohort between these variables has been closely approximated. This implies that, at least in terms of the time-independent variables, the two datasets are very similar.

The time-dependent variables (SES, Loss, Dynamic) are compared in Figure S5, which presents a statistical analysis of these variables between the DANLIFE and synthetic cohorts. The Dynamic variable is closely aligned in terms of its mean, standard deviation, skewness and kurtosis values across the entire time period. This demonstrates that the simulation of the Dynamic ACE closely matches the variable in the DANLIFE cohort. The Loss variable also has very similar mean and standard deviation values, but there are slight differences in skewness and kurtosis values with the DANLIFE data demonstrating a decline across the time period whilst the simulated data is more consistent. Finally, the SES variables have similar skewness and kurtosis values, but there is a greater difference in the mean and the standard deviation values with the synthetic data showing an increase over time whilst the DANLIFE cohort exhibits a decline. Despite these small differences, the closeness of these values overall means that these variables are similar enough to capture the patterns present in the DANLIFE data whilst also prioritising interpretability and the simplicity of the representation.

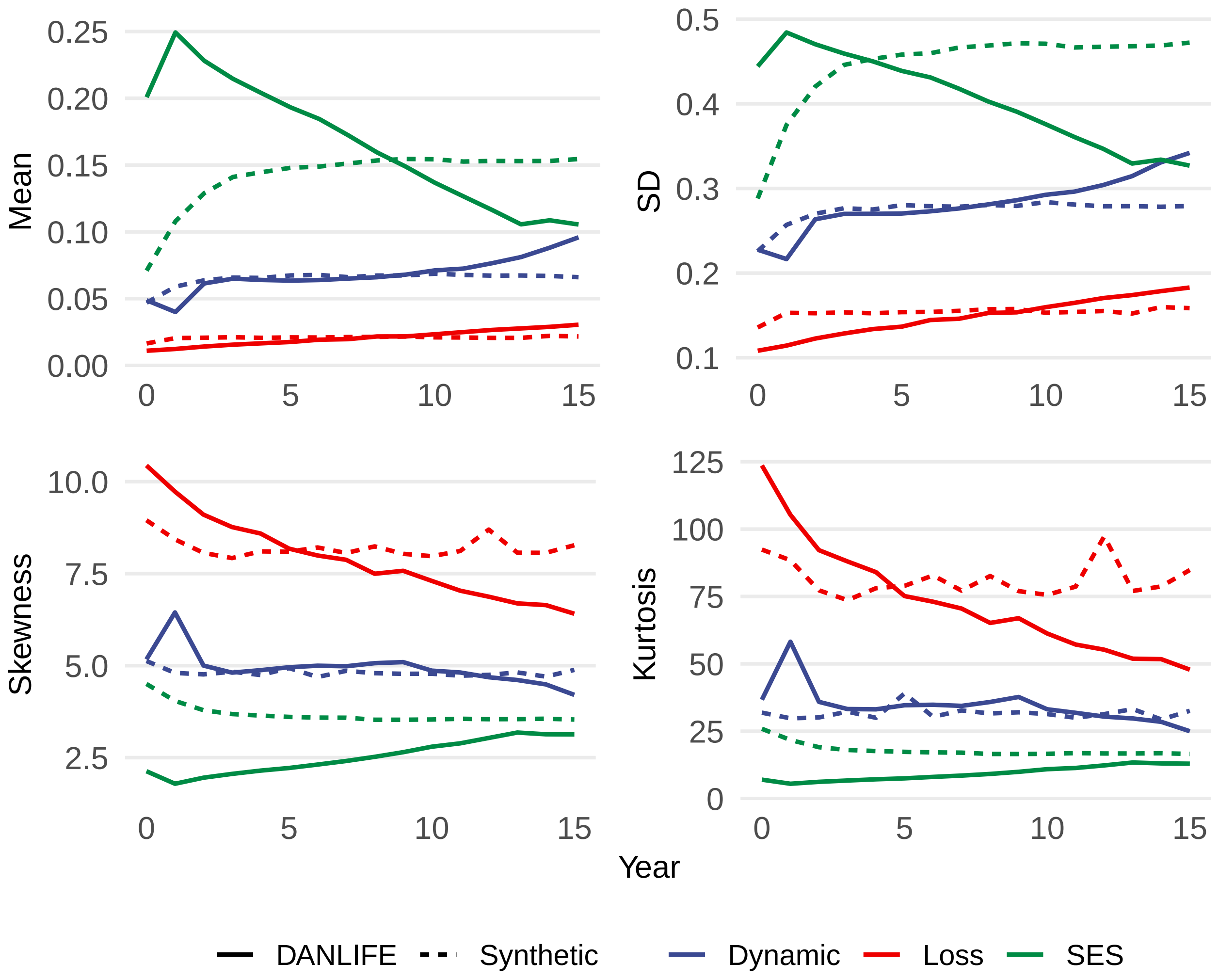

Figure S5. Comparison Between Time-Dependent Variables in the DANLIFE and Synthetic Datasets. The plots show the mean, standard deviation (SD), skewness, and kurtosis over time for the three different time-dependent variables (Dynamic, Loss and SES), with distinct lines representing the DANLIFE and synthetic datasets. Each plot is colour-coded by variable and uses different line types to distinguish between the datasets. The x-axis represents the year, while the y-axis varies according to the measure being plotted.

Outcome

Multiple binary outcomes ($y \in [0,1]^N$) were simulated to represent key life course patterns (LCPs). The outcomes fall into four groups: periods, repeats, order, and timing, which capture different life course patterns. Each group contains multiple simulated scenarios that fall into that category to allow an in-depth exploration of the capability of deep learning (DL) models to learn each life course pattern. The output is binary and designed to correspond to whether an individual is hospitalised with DANLIFE, following the lead of Davies et al.[16]. The different LCPs are identified as follows:

- **Period:** Represents critical and sensitive periods, where outcomes depend on events occurring during certain life stages.
- **Repeats:** Positive outcomes occur when events repeat consecutively over several years.
- **Order:** Examines the sequence of events, where an exposure event must precede another for a positive outcome.
- **Timing:** Focuses on the proximity of events, where multiple exposures occurring close together trigger an outcome.

Rules governing each LCP scenario are detailed in Table 1 in the main text, with percentages of positive outcomes closely matching the 2.58% observed in the real DANLIFE cohort[16]. This low prevalence is typical in medical outcomes and helps ensure realistic testing conditions. To minimise complexity and understand how the models perform in simple representative scenarios, the rules initially only encompass one or two of the temporal variables. In reality, it is likely that the underlying biological and societal mechanisms that give rise to negative health outcomes are considerably more complex than the relationships within this analysis. However, the LCPs are the most simplistic formulations of the hypothesised pathways and consequently can be used in conjunction to form more complex and realistic inter-dependencies between the variables.

To reflect real-world unpredictability, noise was added by randomly flipping the outcomes of 10% of individuals with both positive and negative outcomes in the training set (but not in the test set). This ensures that model performance can be accurately assessed on unseen data without overfitting to noisy training data. The cohort was divided into training and testing sets to provide a fair comparison across models.

Experimental setup

Data Preparation

Before training the DL models, the dataset was preprocessed by normalising the ordinal categorical variables, which ensured that all feature values were on a comparable scale. All data were normalised using the `MinMaxScaler` from the `scikit-learn` package, which was fitted to the training set and then the transform is applied to both training and testing sets. This is not required and therefore, omitted for the XGBoost and logistic regression (LR) models. Normalisation was chosen over standardisation due to the non-normal distribution of the data.

Hyperparameter Optimisation Process

Each of the six models was fitted individually to every LCP from Table 1 in the main text. The models were trained using `tsai`, `xgboost`, `scikit-learn` and `optuna`, and cross-validation was performed to assess model performance[19–21]. Specifically, we used stratified 2-fold cross-validation to ensure robustness of parameter choice and generalisability of the results. The training dataset is split into 2 folds and within each trial the model is trained and validated 2 times, each time a different fold is used as the validation set and the remaining data as the train set. The validation set acts as a test set to assess model performance without using the unseen test set. For 200 trial parameter sets, the model performance, measured with average precision (AP), is averaged across the 2 runs and the final hyperparameter values are chosen as the values from the trial that maximised this value. Each model was fitted to the data three times each time using a different random seed for training and the resulting F1, area under the precision-recall curve (AUPRC), area under the receiver operating characteristic curve (AUROC) and Brier scores are the average values across the three runs.

Logistic Regression

The LR model was trained using a 2D flattened form of the data. The parameter C was optimised from a set of predefined values $[10^{-5}, 10^{-4}, 0.001, \ldots, 10^{5}]$, with the value that maximised the AUPRC score on the validation set selected. In contrast to the DL models, the LR model search space was explored using gridsearch as only a single parameter was optimised. L1 regularisation was used in order to reduce overfitting, and class balancing addressed the imbalanced nature of the dataset. The LR model was written in the `scikit-learn` package version 1.5.1[22].

XGBoost

XGBoost was coded using the `xgboost` package version 2.1.0[20]. The model formulation used the log-loss objective function, and data was flattened to a 2D matrix for input into the model. A range of model hyperparameters were selected using the Tree-structured Parzen Estimator (TPE) sampler and the final parameter values were chosen to maximise the AUPRC score. Key parameters optimised include ‘max_depth‘, ‘eta‘, ‘subsample‘, and others (see Table S2 below). Early stopping was employed to stop training early when an optimum value had been reached. The model also incorporated L1 and L2 regularisation to prevent overfitting. To address class imbalance, the model weights were scaled in accordance with the class distribution.

DL Models

All DL models (ResNet, InceptionTime, MLSTM-FCN, and LSTMAttention) were implemented and trained using the `tsai` python package version 0.3.9[19], with corresponding architectures `ResNetPlus`, `InceptionTimePlus`, `MLSTM-FCNPlus`, `LSTMAttention`, respectively. Each model has a different selection of hyperparameters that govern the model structure and allow it to be adjusted to suit the specific data (Table S3). In addition to the model-specific hyperparameters, further hyperparameters that govern the training process were selected. These include the learning rate that determines the step size in optimisation, the batch size that is the number of data points used within each iteration and the number of epochs which is the number of complete passes through the dataset. Hyperparameters were optimised using the `optuna` package version 3.6.1

Table S2. Hyperparameter tuning search space for XGB. The model parameters that were optimised during hyperparameter optimisation and their corresponding search spaces.

Model	Parameter	Variable	Search Space
XGB	Subsample ratio of columns when constructing each tree	`colsample_bytree`	(0.6, 1.0)
	Learning rate	`eta`	(0.01, 0.3)
	Early stopping patience	`patience`	[5, 10]
	Minimum loss reduction	`gamma`	(0.0, 5.0)
	Maximum depth of a tree	`max_depth`	integer between 3 and 10
	Minimum sum of instance weight required in child	`min_child_weight`	integer between 1 and 10
	Number of trees	`n_estimators`	integer between 100 and 1000
	L1 regularisation term on weights	`reg_alpha`	(0.0, 1.0)
	L2 regularisation term on weights	`reg_lambda`	(0.0, 3.0)
	Subsample ratio of the training instances	`subsample`	(0.5, 1.0)

framework[21], using a Median Pruner to monitors the performance at each epoch and terminate the trial if the performance is deemed suboptimal. This ensures that computational resources are focused on the most promising hyperparameter configurations. The optimisation procedure is formed of multiple trials that each test the model performance for a different set of hyperparameters which are selected from a feature space using the TPE sampler. The Adam optimiser was used along with a maximum of 50 training epochs. These models were tuned using dropout and weight decay (L1/L2 regularisation) to prevent overfitting. We also utilised early stopping to halt model training early if the validation performance did not improve over a set number of epochs. Each model utilised the focal loss function to address class imbalance, which weights the minority class (positive outputs) and more difficult samples greater when calculating the loss to prioritise finding true positives over true negatives. ResNet, LSTMAttention and InceptionTime used GeLU activations, while MLSTM-FCN used ReLU.

Table S3. Hyperparameter tuning search space for DL models. The model parameters that were optimised during hyperparameter optimisation and their corresponding search spaces. The parameters listed under “All DL models" were optimised for all DL models in addition to the model-specific parameters.

Model	Parameter	Variable	Search Space
All DL models	Maximum learning rate	`lr_max`	(1e-4, 1e-1)
	Batch size	`batch_size`	[64, 128, 256]
	Early stopping patience	`patience`	[5, 10]
	Weight decay	`wd`	(0.0, 1e-2)
	Focal loss alpha parameter	`alpha`	(0.1, 0.5)
	Focal loss gamma parameter	`gamma`	(1.01, 3)
MLSTM-FCN	Dropout rate in cells	`cell_dropout`	(0.0, 0.4)
	Number of convolutional layers	`conv_layers`	[[256, 512, 256], [128, 256, 256], [64, 128, 64], [128, 256, 128]]
	Dropout rate in fully connected layer	`fc_dropout`	(0.0, 0.4)
	Number of features in the LSTM hidden state	`hidden_size`	[60, 80, 100, 120]
	Kernel size for each of the convolutional layers	`kss`	[[5, 5, 5], [7, 7, 7], [3, 5, 7], [7, 5, 3], [3, 5, 3]]
	Dropout rate in RNN layers	`rnn_dropout`	(0.0, 0.9)
	Number of RNN layers	`rnn_layers`	[1, 2, 3]
ResNet	Dropout rate in fully connected layer	`fc_dropout`	(0.0, 0.4)
	Kernel size for each convolutional layer	`kss`	[[5, 5, 5], [7, 7, 7], [3, 5, 7], [7, 5, 3], [3, 5, 3]]
	Number of feature maps	`nf`	[32, 64, 128]
InceptionTime	Dropout rate in convolutional layer	`conv_dropout`	(0.0, 0.4)
	Dropout rate in fully connected layer	`fc_dropout`	(0.0, 0.4)
	Kernel sizes for convolutions	`ks`	[20, 30, 40, 50]
	Number of filters per inception block	`nf`	[16, 32, 64, 128]
LSTMAttention	Dimension of the feedforward network model	`d_ff`	[256, 512, 1024, 2048]
	Dropout rate in encoder	`encoder_dropout`	(0.0, 0.4)
	Number of sub-layers in the encoder	`encoder_layers`	[2, 3, 4]
	Dropout rate in fully connected layer	`fc_dropout`	(0.0, 0.4)
	Number of features in the hidden state	`hidden_size`	[64, 128, 256]
	Number of parallel attention heads in self-attention	`n_heads`	[8, 12, 16]
	Dropout rate in RNN layers	`rnn_dropout`	(0.0, 0.4)
	Number of RNN layers	`rnn_layers`	[1, 2]

Calibration

After cross-validation and hyperparameter tuning, the training data is stratified split into two groups, 90% of the data is used to train the final model and the remaining 10% is used to conduct early stopping. Once the final model had been trained then then each of the models was calibrated to improve suitability for risk analysis. The DL models were calibrated using a temperature scaling approach using the quantile method, using the `tsai` package version 0.3.9. The same calibration method was used for the XGB and LR models which was the sigmoid approach coded in the `scikit-learn` package. Then the final threshold for

the model metrics and confusion matrix was selected as the value that maximised the F1-score which balances precision and recall.

Evaluation Metrics

We evaluated model performance on the test set, using F1, AUROC, AUPRC, and Brier score. These metrics, particularly AUPRC and F1, help assess performance on imbalanced datasets. AUROC measures the trade-off between true positive rate (TPR) and false positive rate (FPR), giving insight into overall classification performance[23]. AUPRC focuses on precision and recall, ideal for datasets with rare positive outcomes. The F1 score also seeks to provide an overview of precision and recall performance at a given threshold. The Brier score, calculated as the mean squared error between predicted and actual outcomes, was used to measure calibration. The term calibration describes the level of concordance between the model predictions and the actual observed outcomes, and it is imperative to consider this score particularly in risk modelling. By using these metrics, we ensure a comprehensive evaluation of model performance across all simulated LCPs. Additionally the AP score which is an approximation of AUPRC was used to determine the best parameters in the tuning process.

Explainability

SHAP (SHapley Additive exPlanations) is an XAI method that allows us to explore both local and global feature contributions, enabling us to understand both individual predictions and overall feature contributions. SHAP is a method based on cooperative game theory that assigns each feature an importance value for a particular prediction by calculating Shapley values. Shapley values are derived by considering all possible combinations of features and the marginal contribution of each feature to the model's output. In this study, SHAP values were computed using the `shap` package version 0.46.0 for the ML models used in our analysis[24]. For the DL models, we computed SHAP values using the Gradient SHAP method, which is particularly suited for DL models. The SHAP analysis was performed on the entire test set samples.

Further Model Performance & Explainability Results

Table S4. Evaluation metrics measuring model performance within four LCPs; period, repeats, order and timing. Metric values for all models and LCPs for a calibrated model with a threshold of 0.5, averaged over the three random seeds. Note the F1 score is threshold-dependent and therefore differs from the table in the main text where the threshold has been selected to optimise the F1 scores. The metrics displayed are sensitivity (recall), specificity, precision, F1 score, accuracy, NPV (negative predicted value), AUPRC (area under the precision-recall curve), AUROC (area under the ROC curve) and brier score. LR = logistic regression, XGB = XGBoost, MLF = MLSTM-FCN, IT = InceptionTime, LSTMA = LSTMAttention.

	Model	Sensitivity	Specificity	Precision	F1	Accuracy	NPV	AUPRC	AUROC	Brier
**Period**										
1	LR	99.66	**100.00**	**100.00**	99.83	99.99	99.99	**100.00**	**100.00**	0.07
1	XGB	99.94	99.99	99.78	99.86	99.99	**100.00**	**100.00**	**100.00**	**0.03**
1	MLF	**100.00**	**100.00**	**100.00**	**100.00**	**100.00**	**100.00**	**100.00**	**100.00**	4.10
1	IT	99.94	99.81	94.13	96.94	99.81	**100.00**	99.99	**100.00**	0.25
1	ResNet	95.00	**100.00**	**100.00**	97.32	99.85	99.85	99.94	99.95	0.12

Table S4, continued.

	Model	Sensitivity	Specificity	Precision	F1	Accuracy	NPV	AUPRC	AUROC	Brier
1	LSTMA	**100.00**	**100.00**	**100.00**	**100.00**	**100.00**	**100.00**	**100.00**	**100.00**	1.05
2	LR	89.04	**100.00**	**100.00**	94.15	99.75	99.75	99.96	**100.00**	0.25
2	XGB	99.78	**100.00**	99.93	99.85	99.99	99.99	**100.00**	**100.00**	0.02
2	MLF	99.19	99.98	99.28	99.23	99.97	99.98	99.93	**100.00**	0.03
2	IT	99.04	**100.00**	99.93	99.48	99.98	99.98	99.84	99.92	0.06
2	ResNet	99.85	99.90	96.24	97.95	99.90	**100.00**	99.95	**100.00**	0.09
2	LSTMA	**100.00**	99.99	99.78	**99.89**	**100.00**	**100.00**	**100.00**	**100.00**	**0.01**
3	LR	64.29	99.98	98.05	77.62	99.38	99.39	89.26	99.69	0.50
3	XGB	95.83	99.97	98.17	96.99	99.90	99.93	99.22	99.97	0.12
3	MLF	96.92	98.61	75.85	79.91	98.58	99.95	95.47	99.88	1.17
3	IT	95.34	99.87	92.97	94.04	99.80	99.92	97.65	99.97	0.21
3	ResNet	96.92	**99.99**	**99.19**	98.04	**99.94**	99.95	99.73	99.99	**0.06**
3	LSTMA	**97.72**	99.97	98.51	**98.11**	**99.94**	**99.96**	**99.89**	100.00	**0.06**
**Repeat**										
1	LR	13.31	99.76	49.64	20.98	98.23	98.46	36.87	97.53	1.40
1	XGB	91.12	99.76	87.09	89.06	99.61	99.84	95.81	99.92	0.33
1	MLF	98.77	99.99	99.62	99.19	99.97	99.98	99.87	99.99	0.05
1	IT	97.54	99.15	77.60	83.81	99.12	99.96	98.68	99.97	3.93
1	ResNet	99.91	99.88	94.28	96.93	99.89	**100.00**	99.38	99.99	0.10
1	LSTMA	**100.00**	**100.00**	**99.81**	**99.91**	**100.00**	**100.00**	**100.00**	**100.00**	**0.02**
2	LR	27.37	99.83	68.82	39.16	98.83	99.00	51.52	98.23	0.92
2	XGB	**99.76**	99.97	97.85	98.80	99.97	**100.00**	99.98	**100.00**	0.04
2	MLF	96.35	**100.00**	**100.00**	98.13	99.95	99.95	99.99	**100.00**	0.06
2	IT	96.23	**100.00**	**100.00**	98.06	99.95	99.95	99.84	99.99	0.06
2	ResNet	**99.76**	99.95	96.74	98.22	99.95	**100.00**	99.95	**100.00**	0.05
2	LSTMA	99.64	**100.00**	99.88	**99.76**	**99.99**	99.99	**100.00**	**100.00**	**0.01**
3	LR	14.23	99.80	48.36	21.78	98.69	98.88	30.78	95.91	1.08
3	XGB	99.10	99.96	96.87	**97.97**	**99.95**	**99.99**	**99.68**	**100.00**	**0.05**
3	MLF	85.26	**99.99**	**99.54**	90.99	99.80	99.81	98.67	99.95	0.14
3	IT	66.15	97.37	35.49	40.29	96.96	99.54	97.06	99.92	1.37
3	ResNet	94.87	99.95	96.42	95.40	99.88	99.93	98.45	99.39	0.10
3	LSTMA	**99.36**	99.85	90.17	94.45	99.84	**99.99**	97.15	99.97	0.14
**Order**										
1	LR	16.09	99.09	51.55	24.53	94.37	95.14	44.93	95.67	4.20
1	XGB	99.91	99.61	93.96	96.85	99.63	99.99	98.20	99.93	0.37
1	MLF	99.27	99.66	94.68	96.92	99.64	99.96	99.39	99.63	2.64
1	IT	**100.00**	99.89	98.17	99.07	99.89	**100.00**	**99.99**	**100.00**	2.48

Table S4, continued.

	Model	Sensitivity	Specificity	Precision	F1	Accuracy	NPV	AUPRC	AUROC	Brier
1	ResNet	99.74	**99.97**	**99.45**	**99.59**	**99.95**	99.98	99.95	**100.00**	**0.07**
1	LSTMA	95.28	99.96	99.27	97.14	99.69	99.72	99.80	99.99	0.20
2	LR	7.49	99.62	28.21	11.84	97.79	98.16	24.72	95.94	1.77
2	XGB	84.85	99.71	85.57	85.21	99.42	99.69	90.63	99.79	0.47
2	MLF	91.67	99.94	97.03	94.20	99.78	99.83	97.50	99.44	0.20
2	IT	98.06	**99.98**	**98.89**	98.48	99.94	99.96	99.84	99.99	0.05
2	ResNet	95.54	99.96	97.96	96.68	99.87	99.91	98.97	99.82	0.16
2	LSTMA	**99.58**	99.95	97.71	**98.63**	**99.95**	**99.99**	**99.96**	**100.00**	**0.04**
3	LR	2.59	99.80	19.62	4.57	98.06	98.24	17.12	93.75	1.70
3	XGB	90.46	99.84	91.32	90.88	99.67	99.83	94.34	99.88	0.35
3	MLF	96.20	**100.00**	**99.81**	97.97	99.93	99.93	98.89	99.37	0.08
3	IT	99.63	99.98	99.00	99.31	**99.98**	99.99	**99.98**	**100.00**	0.02
3	ResNet	99.07	99.99	99.26	99.16	99.97	99.98	99.79	99.92	0.03
3	LSTMA	**99.81**	99.99	99.36	**99.59**	**99.98**	**100.00**	99.90	99.99	**0.01**
**Timing**										
1	LR	7.54	99.63	38.97	12.62	96.78	97.12	26.59	93.47	2.67
1	XGB	99.84	99.97	99.09	99.46	99.97	99.99	99.99	**100.00**	0.06
1	MLF	55.52	**100.00**	**100.00**	67.65	98.62	98.60	99.95	**100.00**	0.83
1	IT	99.57	99.99	99.73	99.65	99.98	99.99	99.99	**100.00**	0.07
1	ResNet	77.49	**100.00**	**100.00**	84.17	99.30	99.29	**100.00**	**100.00**	0.43
1	LSTMA	**100.00**	99.99	99.79	**99.89**	**99.99**	**100.00**	**100.00**	**100.00**	**0.01**
2	LR	6.79	99.88	39.59	11.49	98.85	98.97	21.15	95.76	0.99
2	XGB	91.70	99.96	96.51	94.04	99.87	99.91	98.71	99.98	0.16
2	MLF	94.12	**99.99**	**98.91**	**96.45**	**99.92**	99.93	99.40	**99.99**	**0.06**
2	IT	**97.89**	99.46	70.49	80.79	99.44	**99.98**	98.68	99.95	0.42
2	ResNet	94.42	99.97	97.77	95.95	99.91	99.94	**99.70**	**99.99**	0.08
2	LSTMA	94.72	97.90	68.36	71.24	97.86	99.94	75.01	99.47	1.81
3	LR	19.40	99.51	50.36	28.00	97.51	97.97	44.87	98.17	1.83
3	XGB	89.53	99.94	97.46	93.33	99.68	99.73	98.15	99.92	0.31
3	MLF	95.20	**99.97**	98.91	97.00	99.85	99.88	99.21	99.84	0.16
3	IT	**99.20**	**99.97**	**99.01**	**99.10**	**99.96**	**99.98**	**99.80**	99.97	**0.04**
3	ResNet	98.73	**99.97**	99.00	98.86	99.94	99.97	99.67	**99.99**	0.06
3	LSTMA	98.93	4.54	2.59	5.05	6.90	99.40	59.82	97.54	25.05

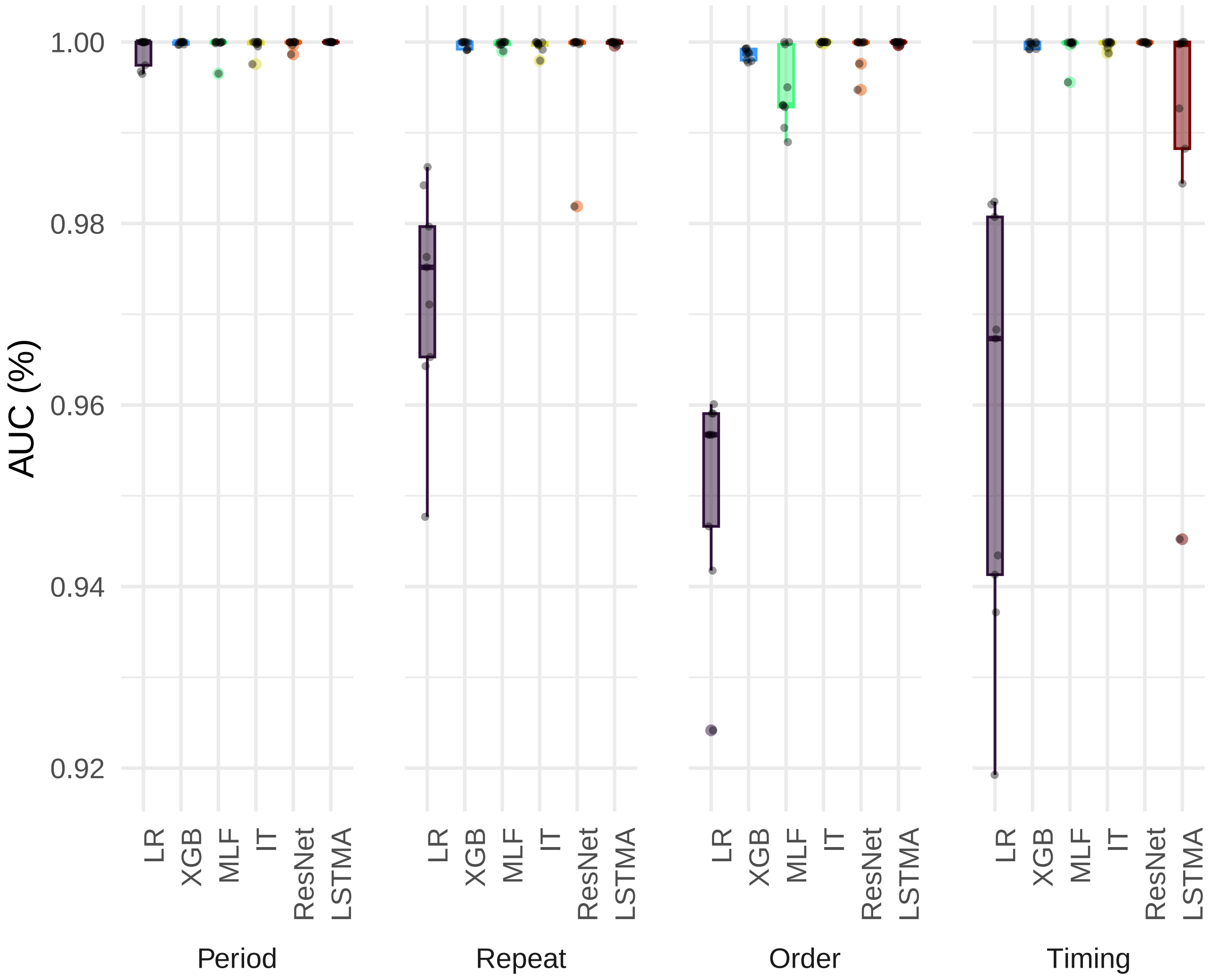

Figure S6. Predictive performance of models for each of the four LCPs (Period, Repeats, Order and Timing). Model performance, measured by AUC scores, shown for each of the three patterns that fall within each LCP. All the AUC scores that resulted in each of the three different seeds used to control model training and hyperparameter optimisation are shown. Boxes show the interquartile range, midpoints depict the median values and whiskers show the minimum and maximum values excluding outliers. LR = logistic regression, XGB = XGBoost, MLF = MLSTM-FCN, IT = InceptionTime, LSTMA = LSTMAttention.

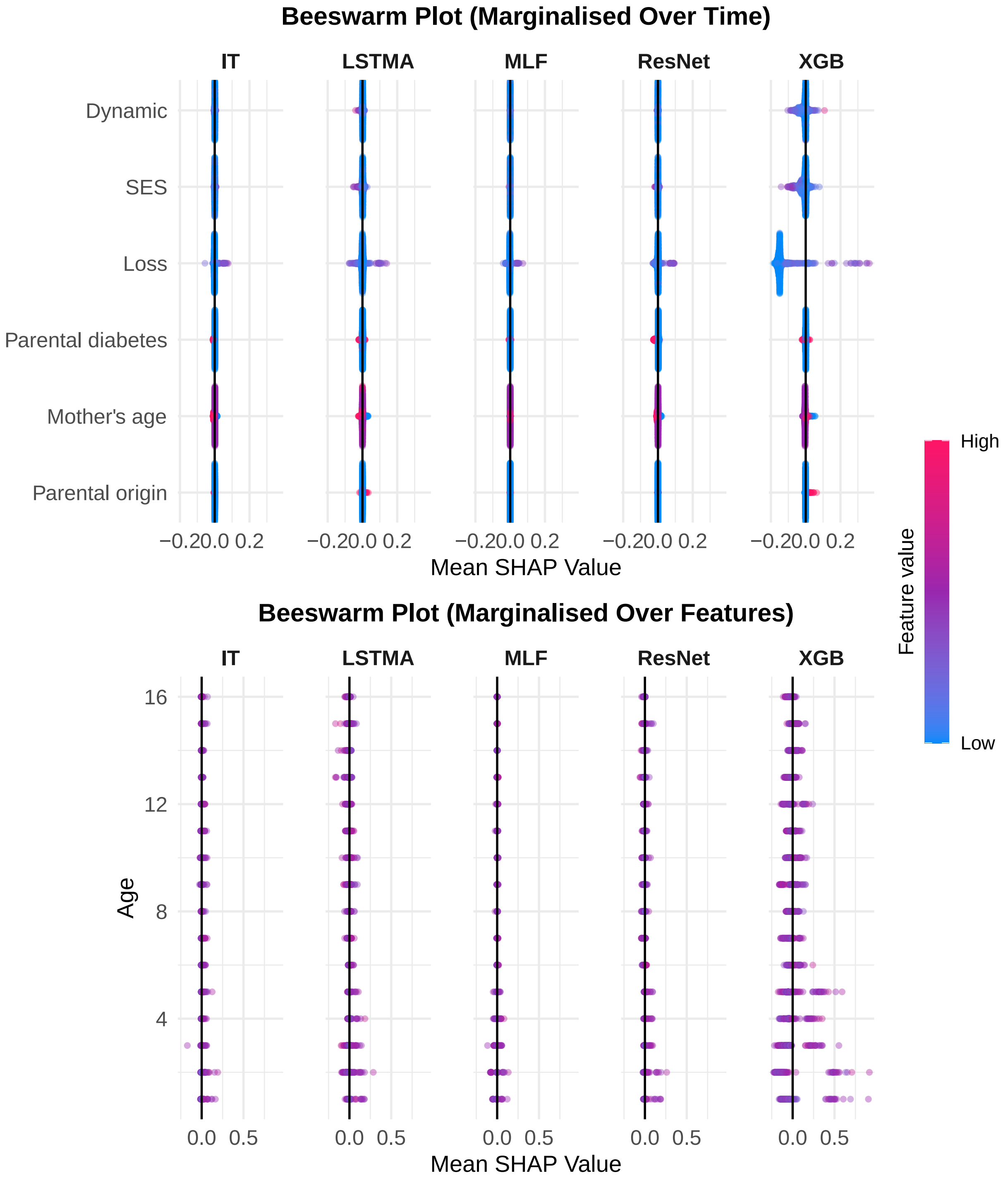

Figure S7. **SHAP Beeswarm Plots for LCP Period3.** The top panel illustrates SHAP values marginalised over time for each feature, grouped by model, with features ordered by mean absolute SHAP values. The bottom panel visualises SHAP values marginalised over features for each age, also grouped by model. Feature values are colour-coded, ranging from low ("Low") to high ("High"). LR = logistic regression, XGB = XGBoost, MLF = MLSTM-FCN, IT = InceptionTime, LSTMA = LSTMAttention.

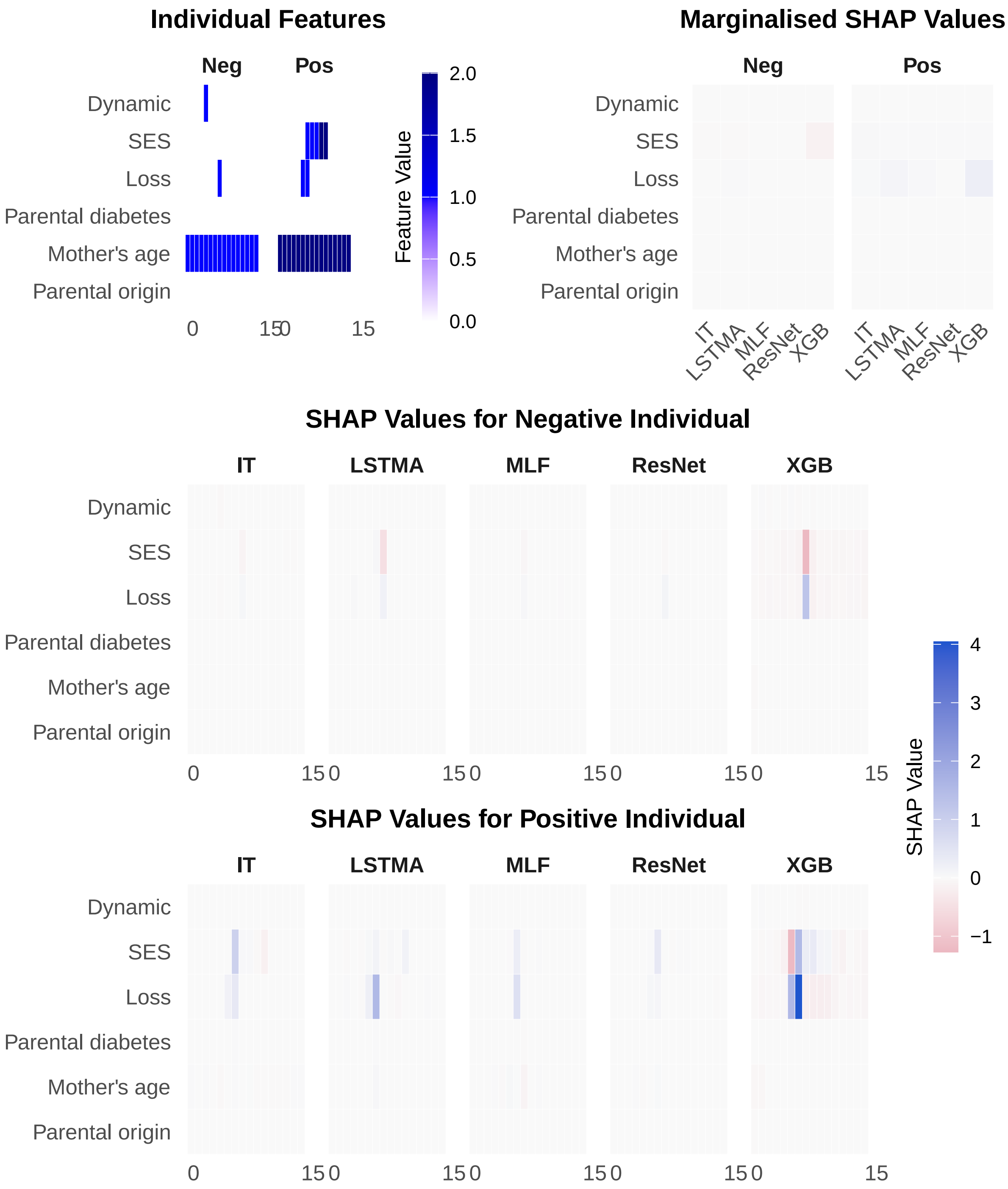

Figure S8. **SHAP Comparison for Individuals for LCP Timing1.** This figure compares SHAP values for two individuals, one positive for the outcome of interest and one negative. The top row includes individual feature heatmaps and SHAP values marginalised over features. The bottom two rows depict the individual-level SHAP values marginalised over time for each individual split by model. SHAP values are shown on a consistent scale across all plots, with colour gradients indicating magnitude and direction (blue to red). LR = logistic regression, XGB = XGBoost, MLF = MLSTM-FCN, IT = InceptionTime, LSTMA = LSTMAttention.

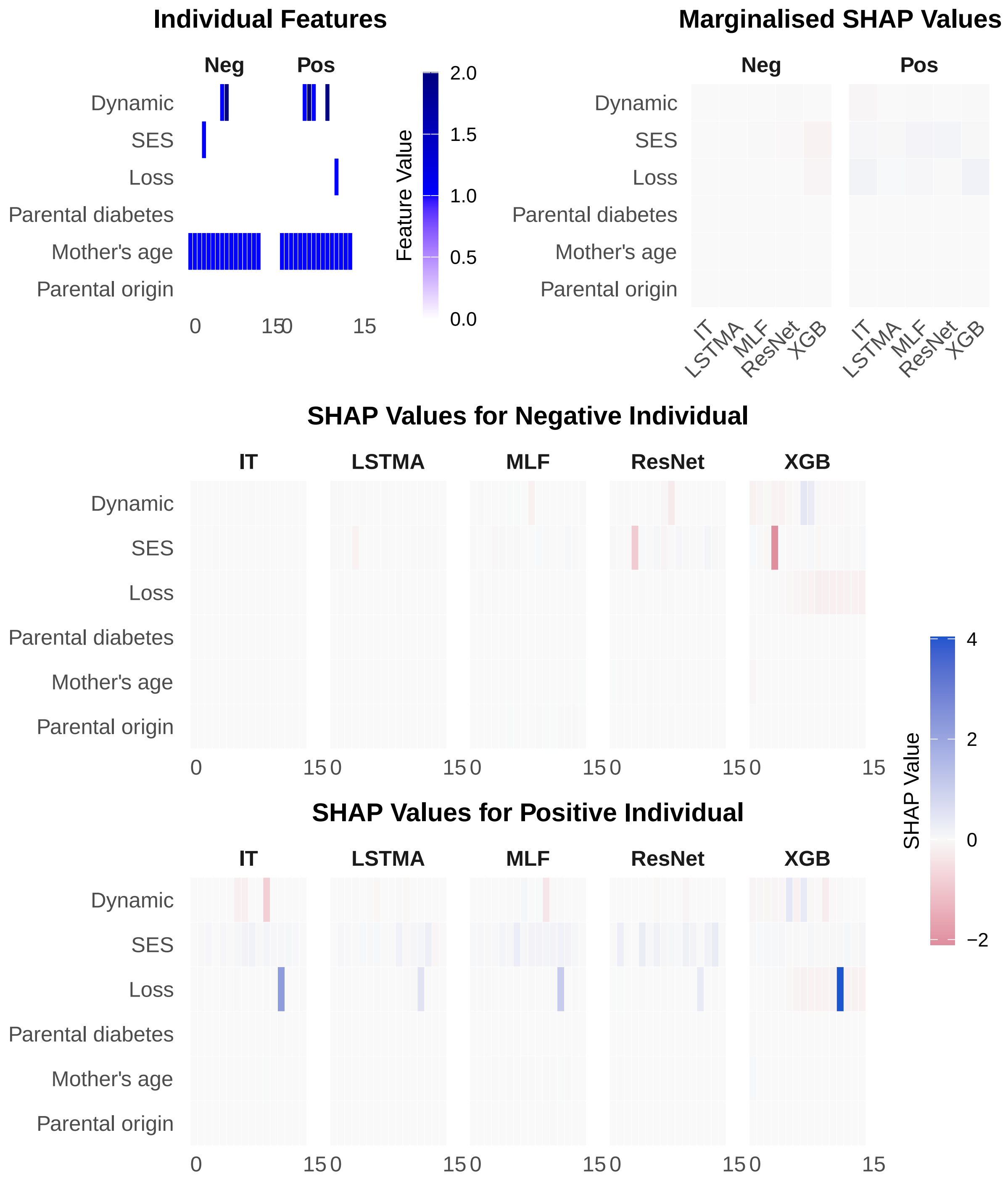

Figure S9. **SHAP Comparison for Individuals for LCP Order3.** This figure compares SHAP values for two individuals, one positive for the outcome of interest and one negative. The top row includes individual feature heatmaps and SHAP values marginalised over features. The bottom two rows depict the individual-level SHAP values marginalised over time for each individual split by model. SHAP values are shown on a consistent scale across all plots, with colour gradients indicating magnitude and direction (blue to red). LR = logistic regression, XGB = XGBoost, MLF = MLSTM-FCN, IT = InceptionTime, LSTMA = LSTMAttention.

References

1. Zerveas, G., Jayaraman, S., Patel, D., Bhamidipaty, A. & Eickhoff, C. A Transformer-based Framework for Multivariate Time Series Representation Learning. *Proc. ACM SIGKDD Int. Conf. on Knowl. Discov. Data Min.* **11**, 2114–2124 (2021).
2. Vaswani, A. *et al.* Attention is All you Need. In Guyon, I. *et al.* (eds.) *Advances in Neural Information Processing Systems*, vol. 30 (Curran Associates, Inc., 2017).
3. Wang, Z., Yan, W. & Oates, T. Time Series Classification from Scratch with Deep Neural Networks: A Strong Baseline. *Proc. IJCNN* **2017-May**, 1578–1585 (2016).
4. Karim, F., Majumdar, S. & Darabi, H. Insights into lstm fully convolutional networks for time series classification. *IEEE Access* **7**, 67718–67725 (2019).
5. Bai, T. & Tahmasebi, P. Attention-based lstm-fcn for earthquake detection and location. *Geophys. J. Int.* **228**, 1568–1576 (2021).
6. Park, J. Y., Lee, K., Park, N., You, S. C. & Ko, J. G. Self-attention lstm-fcn model for arrhythmia classification and uncertainty assessment. *Artif. Intell. Medicine* **142**, 102570 (2023).
7. He, K., Zhang, X., Ren, S. & Sun, J. Deep residual learning for image recognition. *Proc. IEEE Comput. Soc. Conf. on Comput. Vis. Pattern Recognit.* **2016-Decem**, 770–778 (2015).
8. Jung, H. *et al.* Resnet-based vehicle classification and localization in traffic surveillance systems. 934–940 (2017).
9. Xu, W., Fu, Y. L. & Zhu, D. Resnet and its application to medical image processing: Research progress and challenges. *Comput. Methods Programs Biomed.* **240**, 107660 (2023).
10. Lu, Z., Jiang, X. & Kot, A. Deep coupled resnet for low-resolution face recognition. *IEEE Signal Process. Lett.* **25**, 526–530 (2018).
11. Szegedy, C. *et al.* Going deeper with convolutions. *Proc. IEEE Comput. Soc. Conf. on Comput. Vis. Pattern Recognit.* **07-12-June**, 1–9 (2014).
12. Ismail Fawaz, H. *et al.* InceptionTime: Finding AlexNnet for time series classification. *Data Min. Knowl. Discov.* **34**, 1936–1962 (2020).
13. Borra, D., Andalo, A., Severi, S. & Corsi, C. On the application of convolutional neural networks for 12-lead ecg multi-label classification using datasets from multiple centers. *Comput. Cardiol.* **2020-Septe** (2020).
14. Crocker, H. J. & Costall, A. W. An inceptiontime-inspired convolutional neural network to detect cardiac abnormalities in reduced-lead ecg data. *Comput. Cardiol.* **2021-Septe** (2021).
15. Kastrati, A., Plomecka, M. B., Wattenhofer, R. & Langer, N. Using deep learning to classify saccade direction from brain activity. In *ETRA*, 1–6 (2021).

16. Davies, M. *et al.* Childhood adversity and the risk of gestational diabetes: A population-based cohort study of nulliparous pregnant women. *Diabet. Medicine* **41**, e15242 (2024).

17. Sofrygin, O., van der Laan, M. J. & Neugebauer, R. simcausal R package: Conducting transparent and reproducible simulation studies of causal effect estimation with complex longitudinal data. *J. Stat. Softw.* **81**, 1–47 (2017).

18. Rod, N. H., Bengtsson, J., Elsenburg, L. K., Taylor-Robinson, D. & Rieckmann, A. Hospitalisation patterns among children exposed to childhood adversity: a population-based cohort study of half a million children. *The Lancet Public Heal.* **6**, e826–e835 (2021).

19. Oguiza, I. tsai - a state-of-the-art deep learning library for time series and sequential data. Github (2022).

20. Chen, T. & Guestrin, C. Xgboost: A scalable tree boosting system. In *Proceedings of the 22nd ACM SIGKDD International Conference on Knowledge Discovery and Data Mining*, KDD '16, 785–794 (Association for Computing Machinery, New York, NY, USA, 2016).

21. Akiba, T., Sano, S., Yanase, T., Ohta, T. & Koyama, M. Optuna: A next-generation hyperparameter optimization framework. In *Proceedings of the 25rd ACM SIGKDD International Conference on Knowledge Discovery and Data Mining* (2019).

22. Pedregosa, F. *et al.* Scikit-learn: Machine learning in Python. *J. Mach. Learn. Res.* **12**, 2825–2830 (2011).

23. Huang, J. & Ling, C. X. Using AUC and accuracy in evaluating learning algorithms. *IEEE Transactions on Knowl. Data Eng.* **17**, 299–310 (2005).

24. Lundberg, S. M. & Lee, S.-I. A unified approach to interpreting model predictions. In Guyon, I. *et al.* (eds.) *Advances in Neural Information Processing Systems 30*, 4765–4774 (Curran Associates, Inc., 2017).
